# Assessing the potential of bacterial signal peptides for radiopharmaceutical applications

**DOI:** 10.1038/s41598-025-18831-z

**Published:** 2025-09-12

**Authors:** Zukaa Al Taleb, Ina Hierlmeier, Heiko Heilmann, Martin Jung, Mark Bartholomä, Bernd Bufe

**Affiliations:** 1 Department of Informatics and Microsystems Technology, University of Applied Sciences Kaiserslautern, 66482 Zweibrücken, Germany; 2 Department of Nuclear Medicine, Medical Center, PharmaScienceHub (PSH), Saarland University, 66421 Homburg, Germany; 3 Medical Biochemistry and Molecular Biology, Saarland University, 66421 Homburg, Germany

**Keywords:** Target identification, Pharmaceutics, Cancer

## Abstract

Bacterial signal peptides are a large group of high-affinity ligands for formyl peptide receptors (FPRs). These receptors are highly enriched on the surface of glioblastomas and several other tumor types. In this study, we evaluated the potential of bacterial signal peptides, a yet relatively unexplored class of high affinity FPR agonist for radiopharmaceutical applications. We tested a range of bacterial signal peptides and their fluorescently labeled derivatives to identify peptide residues that allow chemical modification without drastic loss of affinity. We then developed a selective fluorescent peptide derivative with more than 1000-fold selectivity towards FPR1, which binds rapidly at low nanomolar concentrations and forms stable receptor-ligand complexes that can persist for up to 72 h. This peptide probe effectively bound to a human glioma cell line U87-MG and efficiently penetrated spheroids derived from U87-MG cells. Finally, we have designed a corresponding metal chelate-peptide conjugate with similar affinity that is taken up by FPR1-transfected HEK293T cells and naturally FPR1-expressing U87-MG cells. A pilot study with planar scintigraphy in healthy mice showed no substantial uptake or retention in healthy organs, suggesting that bacterial signal peptides could indeed be an interesting tool for the development of radiopharmaceuticals.

**Supplementary Information:**

The online version contains supplementary material available at 10.1038/s41598-025-18831-z.

## Introduction

We recently discovered bacterial signal peptides as one of the most complex classes of natural activators of the innate immune system^1^. Currently, only 170,000 bacterial signal peptides are annotated in the signal peptide database, but their real number is likely in the range of several billions^2^. Despite their enormous number and structural heterogeneity, a fairly large amount of the molecules can be detected by formyl peptide receptors (FPRs)^1^. FPRs comprise a small family of chemotactic G-protein-coupled pattern recognition receptors consisting of only three members (FPR1, FPR2 and FPR3) that are capable of detecting a vast array of structurally diverse peptides with high affinity^3,4^. To this end FPRs employ an unusual detection mechanism that likely relies on the recognition of a conserved three-dimensional peptide motif^1^. This enables them to combine structural promiscuity with high specificity and sensitivity and solves the problem of detecting a vast number of distinct sequences yet maintaining selectivity.

This unique mechanism enables FPRs to play a primary role in regulating innate immune responses during bacterial infections by sensing N-formylated signal peptides as metabolites released by bacteria^5^. However, FPRs can also interact with a range of further structurally diverse ligands from different sources such as viruses, host endogenous compounds and a variety of synthetic substances^6–8^. Consequently, FPRs are not only involved in the protection of humans from bacterial and viral infections but they are also involved in the regulation of many other pro- and anti-inflammatory physiological processes^9^ ranging from atherosclerosis^10^ over cancer^11^ and tissue damage^12^ to neurodegeneration^13^. Many studies have shown that FPRs expression is pronouncedly increased in different types of tumors including glioblastoma^14^, ovarian cancer^15^, colon cancer^16^ and bladder cancer^17^. There is clear evidence of an up to 10-fold enrichment of FPR1 in glioblastoma multiforme^18^. Interestingly, the abundance of FPRs in tumor cells combined with their limited occurrence in healthy tissue suggests that targeting these receptors with peptide-based probes may represent a highly selective treatment approach for several types of cancer^19–21^. In addition, studies that investigated the functional effects of FPRs in glioblastoma multiforme showed that the activation of FPR1 by the chemotactic peptide ligand N-formyl-methionyl-leucyl-phenylalanine (f-MLF), affects glioblastoma cell migration and survival, which suggest that FPR1 may contribute to tumor progression and metastasis^22,23^.

Recently, radioligands have become invaluable tools for peptide receptor imaging and therapy^24,25^. These findings already prompted researchers to explore the use of radiolabeled FPR-targeted peptide FLFLF as a new avenue to treat various types of inflammatory disorders. A recent study developed FPR1 antagonist as a nano-radiotracer neutrophil-specific probe for detecting chronic inflammation in atherosclerosis-prone cells in vivo^26^. Mattila et al. investigated the FPR1-targeting peptide FLFLF in Mycobacterium tuberculosis-infected macaques as a neutrophil-specific probe^27^. Yang et al. developed FPR1 probes for activated macrophages to monitor inflammation and evaluate treatment of osteoarthritis in rats^28^. However, several technical obstacles such as hydrophobicity of FLFLF seriously limit its use. Next, the amount of radiotracer reaching the inflammation site is frequently below 1%, which produces very poor target-to-background ratios^29,30^. Thus, further peptide radioligands with improved properties would be of great interest. We previously identified several new bacterial signal peptides as high affinity ligands for FPRs that activate individual FPRs at concentrations ranging from low nanomolar to picomolar^1,2^. Given the large amount of theoretically available peptide sequences with a divergent structure, their high affinity and receptor-subtype selectivity make bacterial signal peptides represent an excellent reservoir for the development of peptide probes for the individual FPRs with improves properties. Moreover, probes targeting FPR1 may foster addition therapeutic strategies for treating aggressive tumors, whose prognosis remains poor despite intense treatment^31–33^. The fact that FPR1 is abundantly expressed in gliomas and other cancer cells in combination with the observation that activation of FPR1 stimulates receptor-mediated endocytosis^34–36^, makes it a highly attractive target for radiopharmaceutical development. Receptor-mediated internalization into tumor cells is often advantageous for radiopharmaceutical applications as this generally results in a prolonged retention of the radiotracer in the tumor. Radioligands targeting FPR1 could provide a unique opportunity to better track FPR1 expression and tumor associated FPR1 activity in vitro and in vivo. Moreover, they offer a potential for future use in diagnostic and therapeutic radiopharmaceutical applications. Given that FPR1 expression is significantly upregulated in various types of cancer cells^38^, we decided to focus on the development of a FPR1-targeting radiopharmaceutical in this work. For this, we first compared the response pattern of serval potent agonists for FPR1 activation and internalization in FPR1-expressing cells. Toward this aim, we identified a novel small molecule, the bacterial signal peptide f-MVPIKI, as an agonist probe of high affinity and selectivity for FPR1. In addition, we developed a potential radiotracer by introducing a metal chelator (DOTA) via a 6-aminohexanoic acid spacer (Ahx) to f-MVPIKI enabling the radiolabeling with the positron-emitter ^68^Ga for diagnostic PET imaging and the beta-emitter ^177^Lu for targeted radioligand therapy applications. We studied this peptide probe with regards to its binding, uptake, stability, and activity as well as its endocytosis triggered internalization. In addition, we investigated the binding and penetration of the peptide probe to U87-MG tumor spheroids in 3D cell cultures as an initial step in translating this peptide-based radiotracer to in vivo applications.

## Results

### Characterization of peptide-ligand derivatives for selective binding and activation of the FPR receptors

**Fig. 1:**
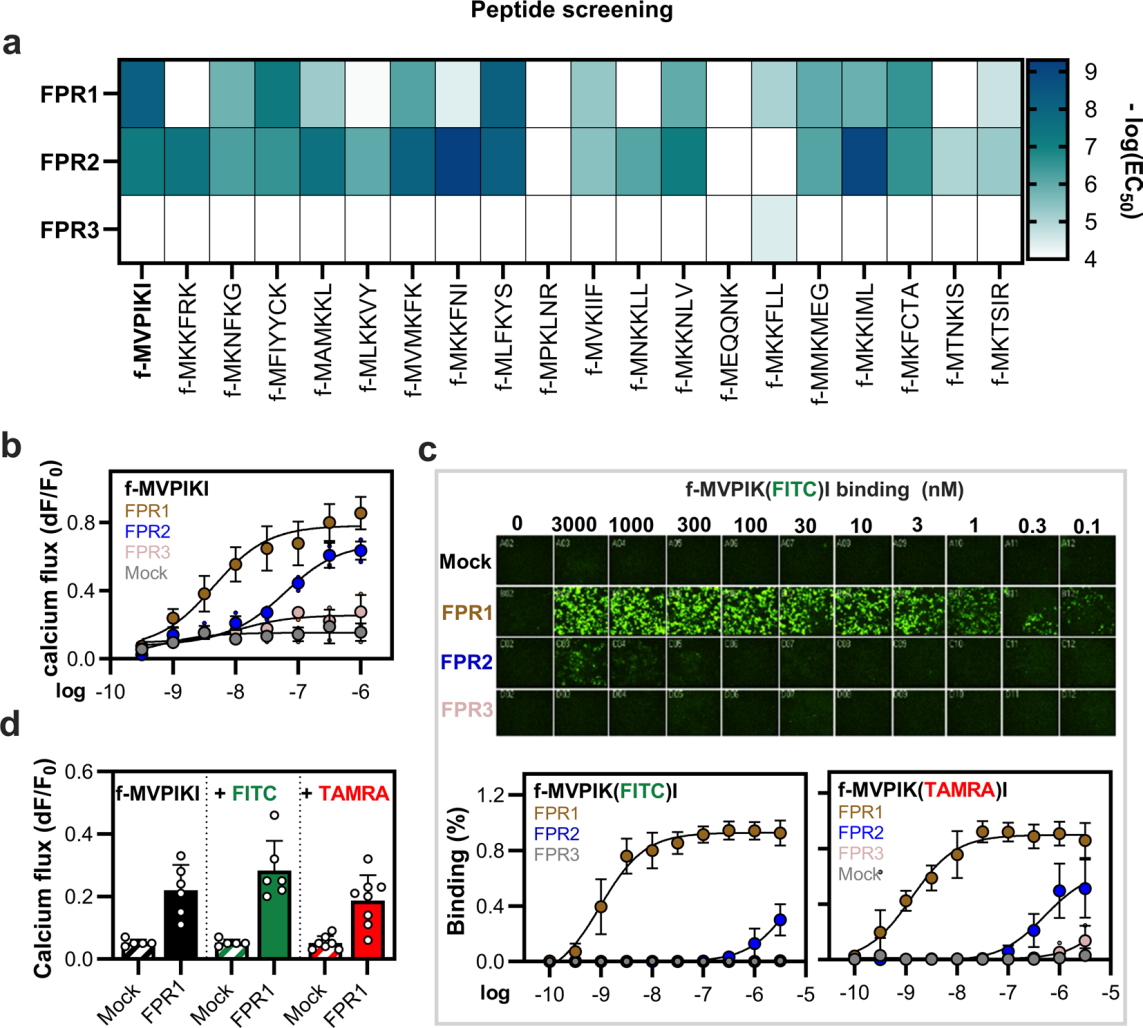
Characterization of formylated peptide ligands for FPR receptor specificity, binding affinity, and calcium mobilization. (**a**) Heatmap displaying the EC_50_ values for peak calcium responses determined through the signal amplitude (dF/F0) of FPR1, FPR2 and FPR3 transfected HEK293T cells after stimulation with different peptide derivatives. Peptide sequences are displayed in the one letter code, f- denotes the formyl group at the N-terminus. (**b**) Concentration-dependent activation of Ca^2+^ mobilization by f-MVPIKI in FPR1 transfected HEK293T cells. Error bars denote SD, *n* = 6; *N* = 6. **c**: Upper panel: representative microscopic images displaying the concentration dependent receptor affinity of the fluorescent labelled f-MVPIK(FITC)I probe bound to FPR1, FPR2, FPR3 and mock transfected HEK293T cells. Lower panel: concentration dependent binding of two different fluorescence labelled peptides f-MVPIK(FITC)I and f-MVPIK(TAMRA)I to HEK293T cells transfected with mock, FPR1, FPR2 or FPR3, respectively. Error bars denote SD, *n* = 3; *N* = 9. **d**: Calcium response of FPR1 transfected HEK293T cells upon stimulation with 1 nM f-MVPIKI, f-MVPIK(FITC)I or f-MVPIK(TAMRA)I display similar sensitivity. Error bars denote SD, *n* = 3; *N* = 6.

In this study, we focused on the development of a radiotracer for FPR1 receptor. To identify a highly sensitive lead peptide sequence, we first investigated the concentration response of 20 sequence divergent formylated peptides to human FPR1 FPR2 and FPR3 (Fig. 1a). High-throughput calcium imaging experiments, in which we used the peptides in up to 10 µM concentrations, allowed the identification of 14 peptides that activated FPR1, 17 activating FPR2 and only one that activated FPR3. The two peptides that displayed the highest affinity towards FPR1 were f-MVPIKI and f-MLFKYS. We decided to focus on the f-MVPIKI peptide because it showed a preference for FPR1, while f-MLFKYS displays a very similar sensitivity to FPR1 and FPR2. The comparison of the concentration responses of f-MVPIKI towards all FPRs showed that it activated FPR1 with an approximately 14-fold preference (EC_50_ = 4.3 ± 2.4 nM) over FPR2 (EC_50_ = 60 ± 0.76 nM) and did not activate FPR3 at any tested concentration (Fig. 1b). Because calcium imaging only indirectly reports relative differences in the binding affinity of a given ligand to a receptor, we next decided to develop fluorescent peptide derivatives that would allow us to directly investigate the interaction of the peptide with the receptors on the cell surface. We first attached a fluorescein isothiocyanate (FITC) dye to the terminal amino group of the lysine residue in the f-MVPIKI sequence. Using confocal fluorescence microscopy, we observed that the derivative f-MVPIK(FITC)I selectively bound to the surface of FPR1 transfected HEK293T cells in a concentration-dependent manner (Fig. 1c). Specific binding of the probe to FPR1 was already observed at 0.1 nM, while binding to FPR2 was only observed above 100 nM and no detectable binding to FPR3 was noted at any tested concentration. Thus, the fluorescence derivative shows a 1000-fold preference of FPR1 over FPR2, which clearly demonstrates that the addition of an FITC group strongly improves the selectivity of the peptide derivative towards FPR1. To investigate if this effect is specific for the chemical structure of FITC or if it depends more on the position of the chemical modification, we next tested the influence of the fluorescent dye by introducing carboxytetramethylrhodamine (TAMRA) at this position. Again, we observed that the fluorescent derivative f-MVPIK(TAMRA)I selectively bound to FPR1 at 0.1 nM, while we only observed binding to FPR2 above 1 µM and no binding to FPR3 (Supplement Fig. 1). Moreover, both derivatives showed very similar K_d_ values of 1.1 ± 0.5 nM and 3.8 ± 2.5 nM for f-MVPIK(FITC)I and f-MVPIK(TAMR)I, respectively (Fig. 1c). To gain insight into the effect of FITC and TAMRA on the capability of f-MVPIKI to activate FPR1, we next examined the Ca^2+^ mobilization of FPR1 transfected HEK293T cells to both fluorescently labelled peptides. Interestingly, the native peptide f-MVPIKI as well as f-MVPIK(FITC)I and f-MVPIK(TAMRA)I exhibited similar FPR1 activity (Fig. 1d). Taken together, these data clearly show that chemical modifications at the epsilon-amino group of the peptide do not drastically affect the binding affinity or receptor activation. Contrarily, these modifications may even enhance receptor selectivity.

### Capability of the f-MVPIK(FITC)I peptide to bind to FPR1 as well as membrane uptake and endocytosis in FPR1-transfected HEK293T cells

**Fig. 2:**
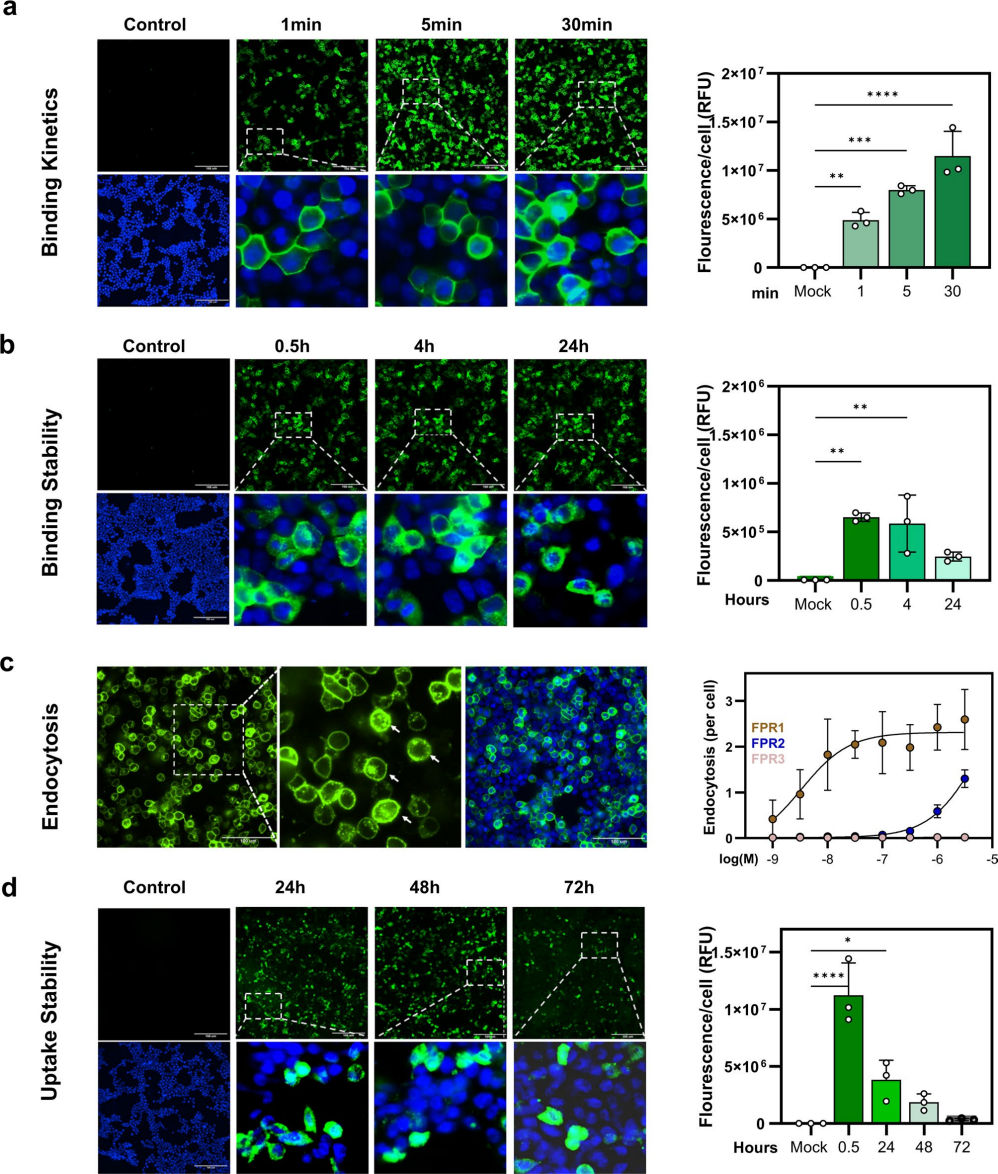
Characterization of the FITC labelled signal peptide for time-dependent binding, stability, endocytosis and uptake. (**a**) Time course of the binding of f-MVPIK(FITC)I to the FPR1 receptor on transiently transfected HEK293T cells. Left upper panel: representative confocal images of the fluorescent peptide only. Left lower panel: magnification of the indicated section displaying the colocalization between the bound peptide and the total cell number using Hoechst 33,342 that stains cell nuclei. Right: mean values of total fluorescence normalized to total cell number. Error bars denote SD, *n* = 3; *N* = 9 (** *P* = 0.005), (*** *P* = 0.0001), (**** *P* < 0.0001). Scale bar = 166 μm. (**b**) Left: Representative binding of f-MVPI(FITC)I to the cell surface of FPR1 transfected HEK293T cells. Right: mean values of total fluorescence normalized to total cell number. Error bars denote SD, *n* = 3; *N* = 9 (** *P* = 0.0002 for 0.5 h) and (** *P* = 0.0002 for 4 h). Scale bar = 166 μm. (**c**) Confocal microscopy images demonstrated that the ligand f-MVPIK(FITC)I triggers FPR1 endocytosis in HEK293T transfected with FPR1. Concentration-endocytosis curves of HEK293T cells transfected with mock, FPR1, and FPR2, respectively. Error bars denote SD, *n* = 3; *N* = 9. Scale bar = 100 μm. (**d**) Uptake of f-MVPIK(FITC) in FPR1 expressing HEK293T cells Living cells were incubated with 1 µM f-MVPIK(FITC) for 0.5 h and then rinsed with 1 × PBS. Images were taken after 24, 48, and 72 h. Error bars denote SD, *n* = 3; *N* = 9 (* *P* = 0.01), (**** *P* < 0.0001). Scale bar = 166 μm.

We next used confocal fluorescence microscopy to investigate the time course of the receptor-ligand interaction, the long-term stability of the receptor-ligand complex, and the uptake of 1 µM f-MVPIK(FITC)I into HEK293T cells transiently transfected with FPR1. Additionally, we aimed to determine the concentration of f-MVPIK(FITC)I required to induce FPR1-mediated endocytosis (Fig. 2). We observed rapid binding of the peptide to FPR1 on the cell surface (Fig. 2a). Within the first minute, approximately 45% of the cells in the visual field were labeled. Longer incubation times of 5 and 30 min resulted in 50% and 60% labeling, respectively. Furthermore, we observed an increase in cytosolic staining over time, which indicates uptake of the peptide into the cells. We next tested the stability of the receptor-ligand complexes formed by the peptide probe (Fig. 2b). For this purpose, HEK293T cells were first incubated at 37 °C for 30 min with 1 µM f-MVPIK(FITC)I. To prevent any further metabolic degradation, the cells were killed by permeabilization with 0.1% Triton X-100 for 20 min at room temperature. The results showed that the binding of the peptide probe remained visible for more than 24 h. During the first 4 h, the fluorescence intensity was comparable to the signal observed after the initial 0.5 h incubation (Fig. 2b). However, at 24 h, the staining intensity decreased to 40% compared to the value obtained at 0.5 h. The observation of increased cytosolic staining during the time-course analysis (Fig. 2a) suggests that the peptides were actively internalized by the cells. To gain further insights into how FPR1 is internalized upon ligand binding, we examined the concentration dependency and selectivity of endocytosis (Fig. 2c). For this purpose, HEK293T cells transfected with FPR1, FPR2, or FPR3 were incubated at 37 °C for one hour with a concentrations range of f-MVPIK(FITC)I ranging from 1 nM to 3 µM. After the incubation period, the cells were washed and analyzed by confocal microscopy. In FPR1-transfected HEK293T cells the peptide probe already induced endocytosis at nanomolar concentrations 4.2 ± 3.7 nM. In contrast, FPR2-transfected HEK293T cells exhibited only minimal endocytosis, which occurred only at higher concentrations 4.5 ± 0.2 µM (Fig. 2c), while no endocytosis was detected in FPR3-transfected HEK293T cells. These results prompted us to investigate the stability of the receptor-ligand complexes in living cells over time, to this end FPR1-transfected HEK293T cells were incubated with 1 µM f-MVPIK(FITC) for 30 min at 37 °C. After 3 times washing with C1 buffer, the cells were further incubated in C1 buffer at 37 °C for an additional 24, 48, and 72 h. The fluorescence intensity gradually decreased over time but remained at 60% after 24 h, 50% after 48 h, and 20% at 72 h, compared to the signal observed at the initial 30 min (Fig. 2d). Taken together these data indicate that the peptide probe f-MVPIK(FITC)I demonstrates rapid binding, triggers FPR1-mediated endocytosis, and exhibits stability of the receptor-ligand complex for more than 24 h.

### Preparation of a FPR1-specific peptide conjugate for radiopharmaceutical applications

**Fig. 3:**
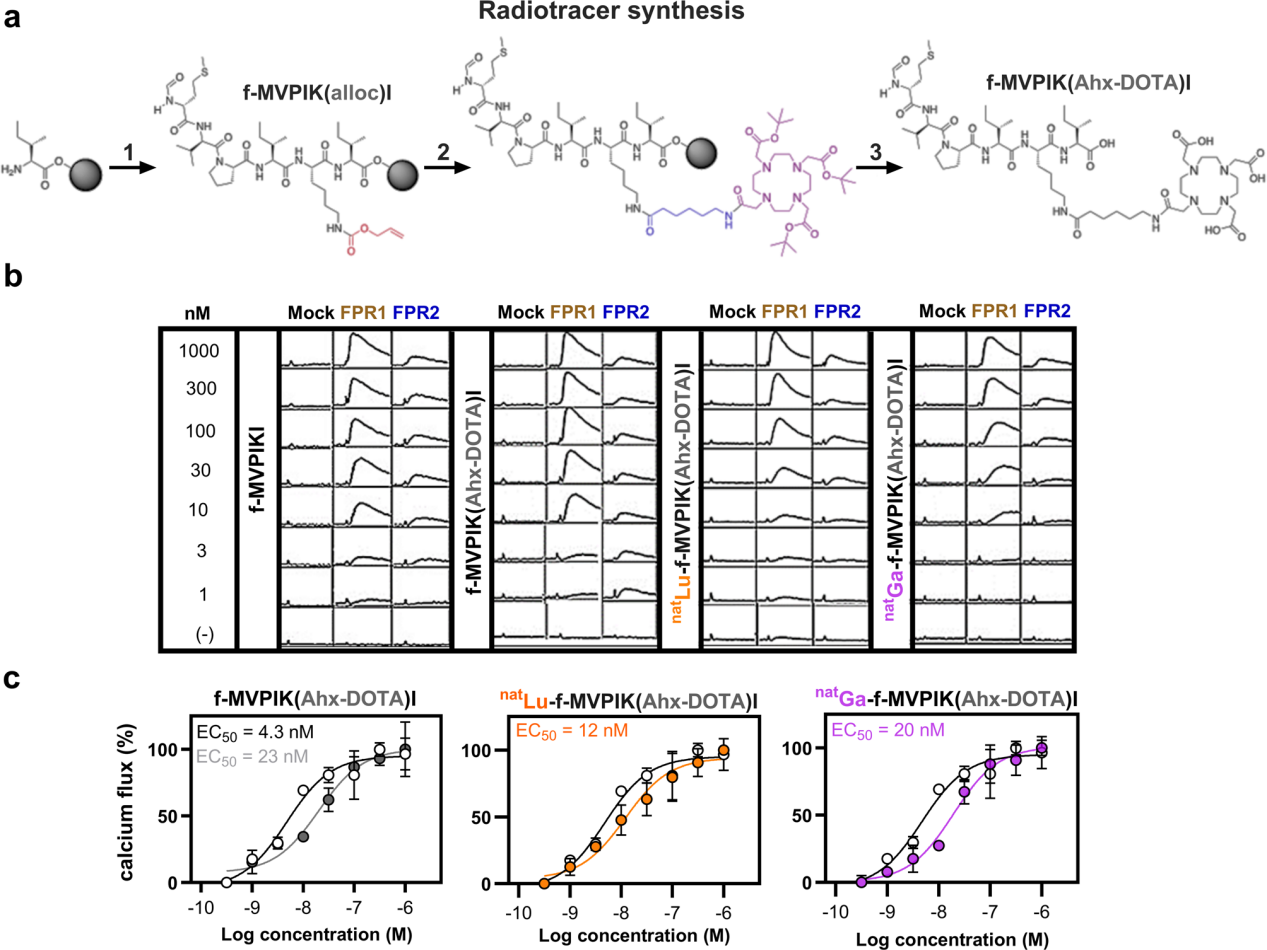
Synthetic strategy for the preparation of the peptide conjugate f-MVPIK(Ahx-DOTA)I and the effect of the modifications on calcium mobilization. (**a**) Solid-phase synthesis of f-MVPIK(Ahx-DOTA)I. Starting from an Ile-Wang resin the amino acids were coupled automatically in a peptide synthesizer followed by manual formylation to obtain the *N*-formylated peptide with an epsilon-alloc protected lysin (1). After removal of the alloc group, the Ahx spacer and tris-*t*Bu protected chelator DOTA were coupled manually (2) followed by a final deprotection and cleavage step (3) to obtain the final conjugate. (**b**) Representative Ca^2+^ signals of HEK293T cells transfected with mock (negative control), human FPR1 or FPR2, after exposure to serial concentrations (from 1 µM to 1 nM) of f-MVPIKI and its modified analogs f-MVPIK(Ahx-DOTA)I, ^nat^Lu-f-MVPIK(Ahx-DOTA)I, or ^nat^Ga-f-MVPIK(Ahx-DOTA)I, (y scale, 1600 relative fluorescent units/square; x scale, 260 s/square). (**c**) Comparison of the concentration-response curves for of f-MVPIKI (black curve, open circles) with its modified analogs f-MVPIK(Ahx-DOTA)I (gray curve, filled circles), ^nat^Lu-f-MVPIK(Ahx-DOTA)I (orange curve, filled circles), or ^nat^Ga-f-MVPIK(Ahx-DOTA)I (purple curve, filled circles) in FPR1 transfected HEK293T cells. Error bars denote SD, *n* = 3; *N* = 9.

The results of the fluorescently labeled f-MVPIKI derivatives showed that chemical modifications at the epsilon amine of the lysine are well tolerated with respect to receptor binding and FPR1-mediated cellular internalization. This prompted us to prepare a peptide conjugate allowing radiolabeling with various metallic radionuclides. For this, we designed the probe f-MVPIK(Ahx-DOTA)I by introducing the versatile metal chelator DOTA (1,4,7,10-tetraazacyclododecane-1,4,7,10-tetraacetic acid) needed for radiolabeling via a 6-aminohexanoic acid spacer (Ahx) to the epsilon amino group of the lysine residue. The peptide was prepared by a combination of automated and manual solid-phase peptide chemistry yielding the final conjugate in an overall yield of 28 ± 6% (*n* = 6) (Fig. 3a). Analysis by low resolution electron spray ionisation mass spectrometry (LR-ESI-MS), high resolution electrospray mass spectrometry (HR-ESI-MS) and analytical HPLC confirmed its identity (*t*_R_ = 10.6 min) and purity (99%). Two radiometals of interest are the positron-emitter ^68^Ga (*t*_1/2_ = 68 min) allowing diagnostic imaging using positron emission tomography (PET) and the beta-emitter ^177^Lu (*t*_1/2_ = 6.7 d), which is extensively used in targeted radioligand therapy. To test the effect of these chemical modifications on the receptor activation in calcium imaging experiments, corresponding non-radioactive ^nat^Ga and ^nat^Lu conjugates were additionally prepared and isolated in 73 and 80% overall yields, respectively. Both compounds exhibited comparable HPLC retention times with 10.6 and 10.7 min for the ^nat^Ga and ^nat^Lu conjugate, respectively. The purity of the ^nat^Ga- and ^nat^Lu-labelled peptide conjugates were 66% and 99%, respectively.

### Mobilization of intracellular Ca^2+^ flux in FPR1 transfected HEK293T cells under stimulation with the peptide conjugate f-MVPIK(Ahx-DOTA)I and its non-radioactive ^nat^Ga and ^nat^Lu complexes

To evaluate the effects of DOTA conjugation and subsequent metal chelation on FPR1 functionality, intracellular calcium flux measurements were performed. Transfected HEK293T cells with mock (negative control), FPR1 or FPR2 were subjected to serial dilutions of the peptide f-MVPIKI, f-MVPIK(Ahx-DOTA)I, ^nat^Lu-f-MVPIK(Ahx-DOTA)I or ^nat^Ga-f-MVPIK(Ahx-DOTA)I, respectively, and peak calcium fluorescence were documented. The maximal amplitudes of the peak calcium fluorescence did not change drastically (Fig. 3b). The EC_50_ of f-MVPIKI in FPR1 transfected HEK293T cells was determined to 4.3 ± 2.4 nM (Fig. 3c). Introduction of the Ahx spacer and the DOTA chelator into f-MVPIKI only moderately affected its ability to trigger Ca^2+^ flux with an EC_50_ = 23 ± 2 nM for f-MVPIK(Ahx-DOTA)I. We next asked whether the corresponding ^nat^Ga and ^nat^Lu complexes of the peptide conjugate f-MVPIK(Ahx-DOTA)I were also able to activate FPR1. Both metalated peptide conjugates activated the receptor and induced the Ca^2+^ signal response with EC_50_ of 12 ± 1 nM for ^nat^Lu-f-MVPIK(Ahx-DOTA)I and 20 ± 1 nM for ^nat^Ga-f-MVPIK(Ahx-DOTA)I (Fig. 3c). Taken together, these data confirmed that the peptide conjugates chelated with ^nat^Ga and ^nat^Lu were able to bind at low nanomolar concentrations and induced a pronounced Ca^2+^ flux in FPR1 expressing HEK293T cells.

### Characterization of peptide probe f-MVPIKI and its derivatives in U87-MG cells

**Fig. 4:**
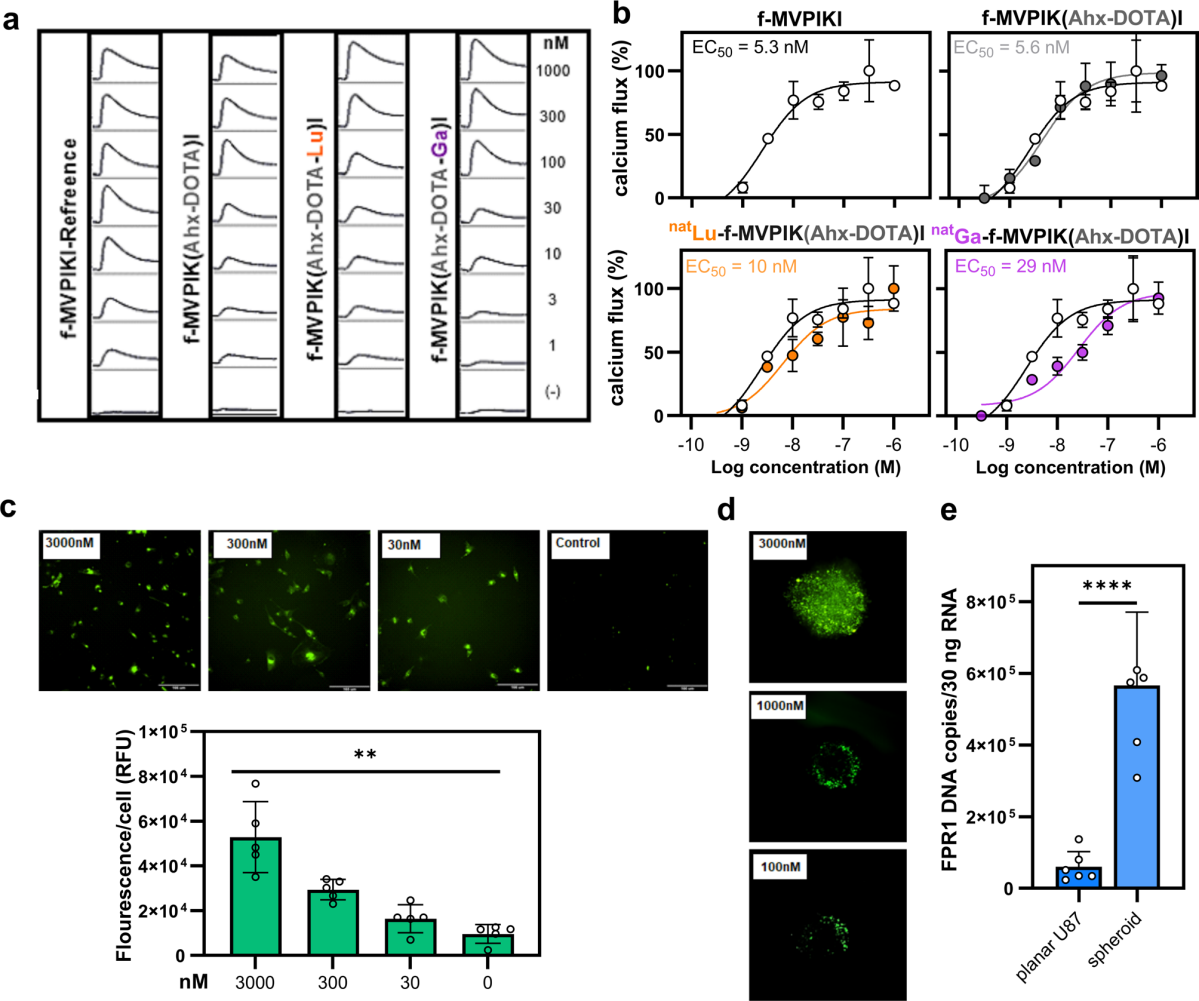
Characterization of the interactions of different conjugate f-MVPIK conjugates with the human malignant glioma cell line U87-MG. (**a**) Representative Ca^2+^ signals of U87-MG after exposure to serial concentrations (from 1 µM to 1 nM) of f-MVPIKI and its modified analogs f-MVPIK(Ahx-DOTA)I, ^nat^Lu-f-MVPIK(Ahx-DOTA)I, or ^nat^Ga-f-MVPIK(Ahx-DOTA)I, (y scale, 2000 relative fluorescent units/square; x scale, 260 s/square). (**b**) Comparison of the concentration-response curves of f-MVPIKI (black curve, open circles) with its modified analogs f-MVPIK(Ahx-DOTA)I (gray curve, filled circles), ^nat^Lu-f-MVPIK(Ahx-DOTA)I (orange curve, filled circles), or ^nat^Ga-f-MVPIK(Ahx-DOTA)I (purple curve, filled circles) on U87-MG cells. Error bars denote SD, *n* = 6; *N* = 12. (**c**) Binding of f-MVPIK(FITC)I to U87-MG cells. Upper panel: Representative fluorescence images of U87-MG cells upon incubation with different concentration f-MVPI(FITC)I for 2 h at 37 °C, scale bar = 100 μm. Lower panel: mean values of total fluorescence normalized to total cell number. Error bars denote SD, *n* = 3; *N* = 9. (** *P* = 0.002). (**d**) The binding of f-MVPIK(FITC)I to U87-MG spheroids. Representative fluorescence images of U87-MG cells upon incubation with various concentration f-MVPI(FITC)I for 24 h at 37 °C. (**e**) RT-qPCR comparing FPR1 expression levels between planar U87-MG cells and spheroids. SD, *n* = 3; *N* = 6 (**** *P* < 0.0001).

We next tested whether the peptide f-MVPIKI and its derivatives could induce intracellular Ca^2+^ release in the human glioblastoma cell line U87-MG that is known to express FPR1, FPR3 and trace amounts of FPR2^39^ (Supplement 2). As expected, stimulation of U87-MG cells with the native peptide f-MVPIKI, the metal-free conjugate f-MVPIK(Ahx-DOTA)I as well as ^nat^Lu-f-MVPIK(Ahx-DOTA)I, and ^nat^Ga-f-MVPIK(Ahx-DOTA)I induced robust Ca^2+^ signals (Fig. 4a). Interestingly, the Ca^2+^ concentration-response pattern of U87-MG cells to f-MVPIKI and its modified analogs was very similar to that obtained for FPR1 transfected HEK293T cells. The peptide f-MVPIKI showed a concentration response of EC_50_ = 5.3 ± 2.4 nM, f-MVPIK(Ahx-DOTA)I activated the receptor with an EC_50_ = 5.6 ± 0.9 nM and the corresponding ^nat^Lu and ^nat^Ga peptide conjugates were able to induce a pronounced Ca^2+^ flux in U87-MG cells with EC_50_ = 10 ± 0.9 nM and 29 ± 2.1 nM, respectively (Fig. 4b). We hypothesized that f-MVPIKI will be able to bind to FPR1-expressing U87-MG cells. To test this, we incubated U87-MG cells with different concentrations of f-MVPIK(FITC)I for 2 h. Confocal microscopy analysis showed that the peptide probe f-MVPIK(FITC)I binds to U87-MG cells in a concentration-dependent manner (Fig. 4c). However, we also noted that the total amount of fluorescence intensity was drastically lower in U87-MG cells than that in FPR1 transfected HEK293T cells indicating that the overexpression system produces larger amounts of FPR1 (compare Figs. 2a and 4c). To establish the capability of f-MVPIK(FITC)I to penetrate a tumor environment, binding of f-MVPIK(FITC) to U87-MG spheroids was tested in a 3D culture model. Life cell analysis using the Incucyte S3 system showed that U87-MG cells were able to form spheroids after five days by incubation at 37 °C in collagen as 3D matrix (Supplement. 3). After incubation of U87-MG spheroids for 24 h with f-MVPIK(FITC), we found that 100 and 300 nM concentrations successfully labelled the cells close to the surface of U87-MG spheroids. At 3 µM, the probe was able to completely penetrate U87-MG spheroids of a diameter of up to 100 μm (Fig. 4d). We also observed a more intense staining of the spheroids in comparison to planar cultures. In line with this observation, RT-qPCR results showed that U87-MG spheroids displayed an approximately tenfold higher level of mRNA-FPR1 compared to planar U87-MG cells (Fig. 4e).

### The peptide conjugate f-MVPIK(Ahx-DOTA)I labels with ^68^Ga or ^177^Lu, shows sufficient stability in vitro, and binds specifically to FPR1 transfected HEK293T and U87-MG cells

**Fig. 5:**
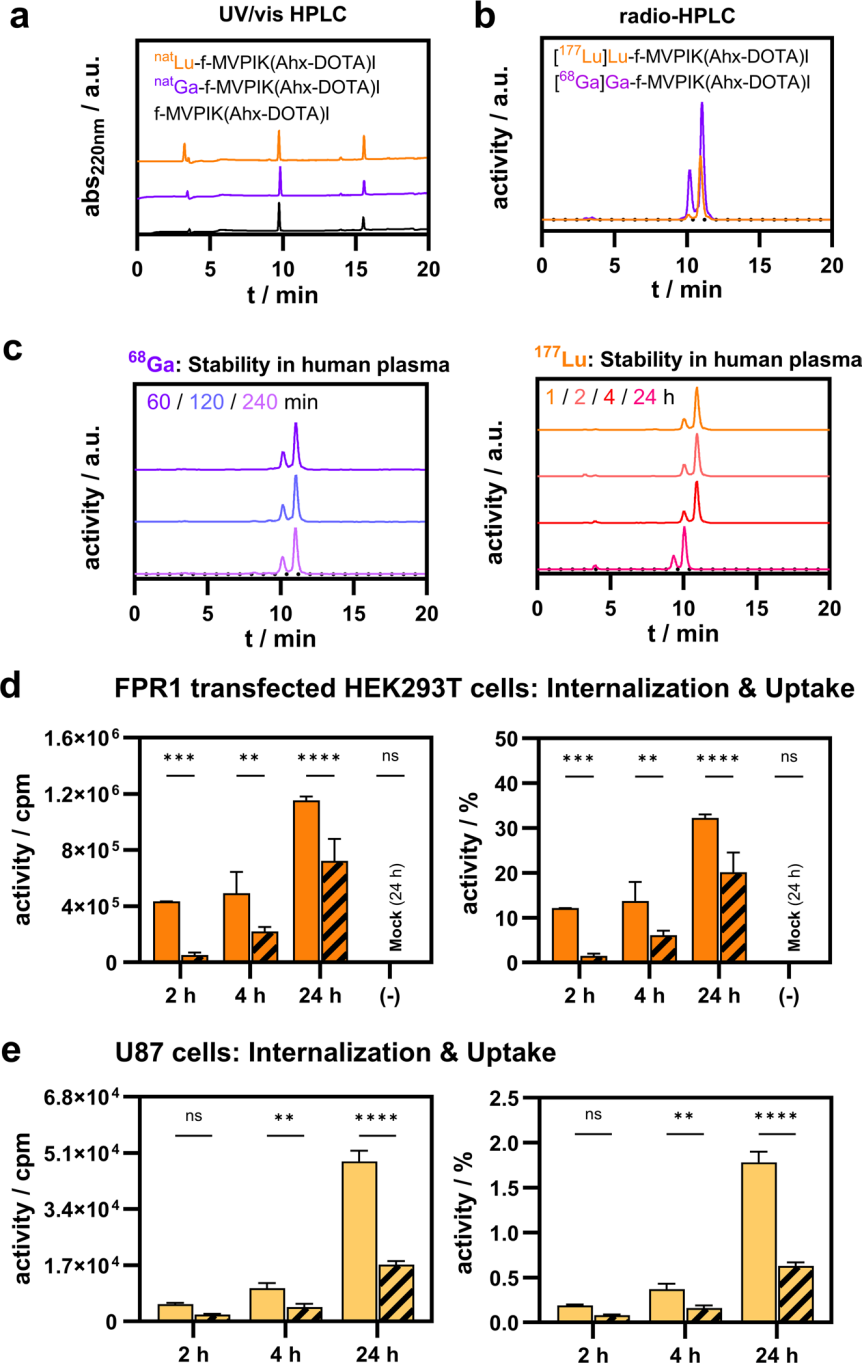
Metabolic stability and cellular internalization of radiolabeled peptide. (**a**) Representative UV/vis-RP-HPLC chromatograms of the bioconjugate f-MVPIK(Ahx-DOTA)I and its ^nat^Ga- and ^nat^Lu complexes. (**b**) Representative radio-RP-HPLC chromatograms of [^68^Ga]Ga-f-MVPIK(Ahx-DOTA)I and [^177^Lu]Lu-f-MVPIK(Ahx-DOTA)I. (**c**) Stability of [^68^Ga]Ga-f-MVPIK(Ahx-DOTA)I and [^177^Lu]Lu-f-MVPIK(Ahx-DOTA)I in human plasma assessed by radio-RP-HPLC, normalized. (**d**) Cellular internalization of [^177^Lu]Lu-f-MVPIK(Ahx-DOTA)I in transfected HEK293T cells over 24 h, total counts (in counts per minute, cpm) and percentage of applied activity. (**e**) Internalization of [^177^Lu]Lu-f-MVPIK(Ahx-DOTA)I in U87-MG cells over 24 h, total counts (cpm) and percentage of applied activity. The striped columns represent blocking experiments in the presence of the specific FPR1 antagonist BOC-FLFLF.

The conjugate was successfully labeled with ^68^Ga in sodium acetate buffer pH 4.0 after incubation for 15 min at 95 °C in quantitative radiochemical yields with molar activities of 25 ± 5 MBq nmol^− 1^ (*n* = 3). Similarly, quantitative labeling was obtained for ^177^Lu using ammonium acetate buffer pH 5.4 in molar activities of 170 ± 10 MBq nmol^− 1^ (*n* = 12). Representative radio-RP-HPLC chromatograms are given in (Fig. 5a) with retention times being in concordance to those of the non-radioactive reference compounds. Partial oxidation of the methionine was noted with the oxidized peptide conjugate eluting just prior the product peak.

Since peptide-based radiotracers can undergo degradation by peptidases, the metabolic stability of radiolabeled f-MVPIK(Ahx-DOTA)I was assessed in complete cell growth medium (MEM + 10% FBS) and human plasma. No change was noted for up to 4 h with the ratio between the tracer and its oxidized form remaining constant (~ 75:25%) (Fig. 5b-c). The absence of any other signals confirmed sufficient stability of the radiotracer. Additionally, the stability was also assessed in human plasma. The ^68^Ga- and ^177^Lu-labeled conjugates were stable for at least 4 h in human plasma. At 24 h, the ^177^Lu-labeled conjugate was completely oxidized, which can be attributed to the experimental conditions (open vial in air). Partial degradation was also observed after 24 h incubation time with a more hydrophilic fragment (24%) eluting just prior to the oxidized conjugate.

Next, FPR1-mediated cell internalization of the ^177^Lu-labeled f-MVPIK(Ahx-DOTA)I) was studied for up to 24 h in FPR1 tranfected HEK293T and U87-MG cells (Fig. 5d-e). In FPR1 tranfected HEK293T cells, uptake increased from 12.11 ± 0.06% at 2 h to 32.25 ± 0.76% of total applied activity at 24 h. FPR1 specificity was confirmed by incubation with a 1000-fold excess of the FPR1 blocking agent BOC-FLFLF, which considerably reduced the cell-bound activity at all time points. The uptake in the blocking group also increased from 1.48 ± 0.49% to 20.16 ± 4.40%. This may be explained by recycling of the receptor within the 24 h time frame of the experiment.

In U87-MG cells, a similar trend was observed with the cell-bound activity increasing from 0.19 ± 0.01% at 2 h to 1.78 ± 0.12% at 24 h. Additionally, no uptake was observed for experiments using FPR1 transfected HEK293T cells in the Mock control confirming receptor-mediated uptake. In general, the uptake in U87-MG cells was lower compared to FPR1 transfected HEK293T cells.

### Radiolabeled [^177^Lu]Lu-f-MVPIK(Ahx-DOTA)I clears rapidly from healthy mice

**Fig. 6:**
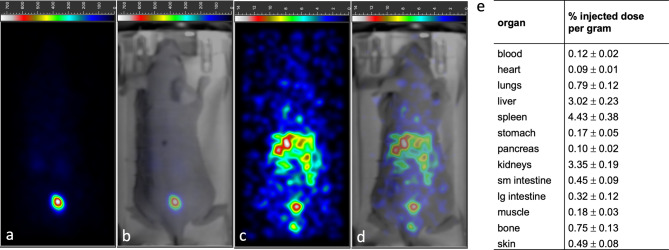
Representative planar scintigraphy scans (**a**,**c**) and combined optical/scintigraphy images (**b**,**d**) of a healthy mouse at 5 min and 60 min post-injection of [^177^Lu]Lu-f-MVPIK(Ahx-DOTA)I conjugate. The tracer is rapidly excreted via the renal pathway with the majority of the activity already being in the bladder after 5 min. No substantial uptake was noted in healthy organs. Of note, the scale bars between the scans at 5 and 60 min are different due to the different amounts of activity remaining in the mouse. (**e**) Ex vivo biodistribution data of [^177^Lu]Lu-f-MVPIK(Ahx-DOTA)I in healthy mice (*n* = 3) at 60 min post-injection.

The biodistribution of the ^177^Lu-labeled peptide conjugate was preliminary assessed by planar scintigraphy imaging and ex vivo biodistribution studies in healthy mice (Fig. 6). In the scintigraphy images at 5 min post-injection (p.i.), the majority of the administered activity was found in the bladder indicating rapid renal clearance of the peptide conjugate. At 60 min, only minimal residual activity was noted in healthy mice with no considerable physiological uptake in other organs, except the excretory organs. The results of the imaging study were confirmed by those of the ex vivo biodistribution experiment. At 60 min p.i., uptake was very low (< 1%) in all organs, except the spleen, the liver, and the kidneys with 4.43 ± 0.38% injected activity per gram, 3.02 ± 0.23, 3.35 ± 0.19% injected activity per gram (%IAg^− 1^), respectively.

## Discussion

In summary our data on the affinity, stability and penetration capability together with the lack of retention in healthy organs demonstrate that bacterial signal peptide derivatives are indeed a promising source for the development of radiopharmaceuticals for imaging and therapeutic applications. In this study, we successfully modified a bacterial signal peptide to develop and characterize the FPR1-selective radiotracer probe f-MVPIK(Ahx-DOTA)I.

Based on the fact that the FPR1 receptor is highly expressed on a variety of cancer cell types but relatively less expressed in normal tissue, FPR1 represents an attractive target for cancer imaging and therapy. In line with this our data confirmed a relatively high FPR1 expression in U87-MG cells at both mRNA and protein levels by RT-qPCR and immunofluorescence (Supplement.2), which is consistent with early reports providing evidence that high FPR1 receptor expression has been considerably detected in glioblastoma cell cancer^18,40^. Based on the knowledge that FPR receptors expressed on the cell surface can be activated by specific agonist binding, and following previous reports showing that activation of FPR triggers GPCR-mediated signaling cascades leading to intracellular Ca^2+^ mobilization^41–43^, we screened an array of bacterial signal peptides for their activation of intracellular Ca^2+^ release in FPR transfected HEK293T cells. This way, we identified the bacterial signal peptide f-MVPIKI as a new target molecule for FPR1, which induces high intracellular Ca^2+^ flux with EC_50_ = 4.3 nM (Fig. 1b). In a next step, we chemically modified the peptide at the epsilon amine group of the lysine with the two different fluorescent dyes FITC and TAMRA to study the cellular fate of these conjugates by fluorescence microscopy and to evaluate the impact of these structural modifications on their binding properties. In consistence with a previous report showing that modification of FPR ligands with fluorescent markers at certain residues do not necessarily alter their affinity or binding properties to the target^36^, we found that modification of the f-MVPIKI peptide with FITC and TAMRA at the lysin had no effect on its binding properties to FPR1 and its ability to induce Ca^2+^ signal (Fig. 1c-d). Notably, our study revealed that the f-MVPIK(FITC)I probe exhibited high affinity, specific and stable binding to the FPR1 receptor in FPR-transfected HEK293T cells, with a dissociation constant K_d_ = 1.1 ± 0.5 nM (Fig. 1c). Our results also showed that the interaction of the f-MVPIK(FITC)I ligand with the receptor on U87-MG cells was slower and less efficient compared to transfected HEK293T cells. In U87-MG cells, the f-MVPIK(FITC)I ligand demonstrated concentration-dependent binding, ranging from 3 µM to 30 nM only after a 2 h incubation period (Fig. 4c). In contrast, 1 µM of the f-MVPIK(FITC)I ligand exhibited rapid binding affinity to FPR1-transfected HEK293T cells, with approximately 45% of the cells in the visual field labeled within the first minute (Fig. 2a). Thus, there is a difference in the binding behavior of the f-MVPIK(FITC)I ligand between naturally FPR1-expressing U87-MG cells and FPR1-transfected HEK293T cells. One possible explanation is that in naturally FPR1-expressing U87-MG cells, the receptor may be distributed compared to the overexpressing HEK293T cells, being present both on the plasma membrane and intracellularly, thus reducing the immediate availability of receptors for ligand binding. Of note a similar mechanism has been already proposed for mouse fpr3^44^. However, a number of alternative mechanisms can also account for the difference. For example, the coexpression of FPR1 with FPR3 in U87-MG cells^39^ may lead to receptor multimerization, which in turn could cause an altered ligand binding behavior. Another possibility is that gene polymorphisms, different G-protein subunits or other receptor associated cofactors such as MARCO or RAGE may lead to alterations in the receptor conformation^45^. The binding results of our peptide probe to the FPR1 receptor and to U87-MG cells encouraged us to synthesize a peptide conjugate that could be used as a radiotracer by introducing the metal chelator DOTA via an 6-aminohexanoic spacer. To also assess the influence of the radiometal cation, the corresponding ^nat^Ga and ^nat^Lu complexes were prepared and tested. Interestingly, neither the introduction of the DOTA chelator nor metal complexation affected the ability to induce of Ca^2+^ flux in FPR1 transfected HEK293T cells with EC_50_ values being in the nanomolar range similar to those of the native peptide f-MVPIKI. Furthermore, our study showed that the bacterial signal peptide probe f-MVPIKI significantly induces Ca^2+^ flux in U87-MG cells. The Ca^2+^ response signal in U87-MG cells that naturally express FPR1 was comparable to that obtained in FPR1 transfected HEK293T cells. These findings are consistent with previous reports showing that FPR1-expressing glioblastoma cells exhibit calcium flux when stimulated with mitochondrial FPR1 peptide ligands^46^. In addition, we observed that the peptide conjugate f-MVPIK(Ahx-DOTA)I and corresponding ^nat^Ga and ^nat^Lu complexes were able to stimulate Ca^2+^ flux in U87-MG cells in nanomolar concentration range. Interestingly, the peptide conjugates did not cause any noticeable shift in Ca^2+^ signaling induction compared to the native peptide probe, neither in FPR1-transfected HEK293T cells nor in U87-MG cells. This indicates that the structural modifications of the peptide conjugates, such as the addition of fluorescent dyes or metal chelators, did not alter the ability of the probe to activate the FPR1 receptor. Additionally, we observed that the uptake of the ^177^Lu-labeled conjugate in U87-MG cells was lower compared to the uptake in FPR1-transfected HEK293T cells. This difference in uptake between the two cell types could be attributed to the lower expression levels of the FPR1 receptor in U87-MG cells compared to the overexpression in FPR1-transfected HEK293T cells. Since the biological behavior of tumors is more accurately represented in 3D tumor spheroid models^47^, we investigated the formation of U87-MG spheroids in 3D cell cultures to improve the in vivo applicability of our results. Remarkably, our FPR1 ligand probe f-MVPIK(FITC)I demonstrated successful binding and penetration into the 3D U87-MG culture model. In this regard, we examined the mRNA expression levels of FPR1 in U87-MG spheroids and found that FPR1 expression was significantly higher in U87-MG spheroids compared to monolayer U87-MG cells (Fig. 4e). This finding suggests that U87-MG tumor spheroids, with their high FPR1 mRNA expression, could serve as an ideal platform for the evaluation of FPR1-specific radiotracers prior to xenograft animal models, facilitating future imaging applications. The metabolic stability of radiolabeled f-MVPIK(Ahx-DOTA)I in both complete cell growth medium and human plasma supports its future use as a novel radiotracer for in vivo applications. In addition, our results confirmed that the radiotracer induces receptor-mediated endocytosis at nanomolar concentrations, confirming its ability to be internalized by target cells. Furthermore, FPR1-mediated cell internalization of ^177^Lu-labeled f-MVPIK(Ahx-DOTA)I) in FPR1-transfected HEK293T and in U87-MG cells was noted for up to 24 h. Of note, no significant uptake of the tracer was detected in healthy organs of healthy mice by planar scintigraphy and in ex vivo biodistribution studies. Additionally, the radiotracer was rapidly excreted via the kidneys, indicating favorable pharmacokinetics. In summary, the high selectivity and binding affinity to FPR1, metabolic stability, and cellular uptake of the f-MVPIK(Ahx-DOTA)I peptide conjugate make it compound a promising candidate and a valuable tool for visualizing FPR1-expressing tumors by nuclear imaging techniques in the future. However, evaluation in vivo in corresponding animal models is thus warranted.

## Materials and methods

All human FPR genes were cloned as described previously^2^. Ultrapure water was obtained from an Aqua-Lab Connect Bio (Aqua-Lab GmbH, Höhr-Grenzhausen, Germany). Gallium-68 was eluted from a Galli-Eo^TM^-generator (IRE, Fleurus, Belgium) or an GalliaPharm^®^-generator (Eckert & Ziegler, Berlin, Germany) operated according to the manufacturer’s instructions. Lutetium-177 was obtained from Monrol (Istanbul, Turkey). For low resolution mass spectrometry (+)-LR-ESI-MS, an Advion expression CMS system (Advion, Ithaca, NY, United States) was used. Matrix assisted laser desorption ionisation coupled with time-of-flight (MALDI-TOF) was used to record high resolution mass spectra by the mass spectrometry service at the department of chemistry of saarland university. High performance liquid chromatography (HPLC) was performed on an Agilent 1260 Infinity System (Agilent Technologies, Santa Clara, CA, Unites States) equipped with an Agilent 1200 UV detector (UV detection at 254 nm) and a FlowStar2 LB 514 radiation detector (Berthold Technologies, Bad Wildbad, Germany) in series. A Phenomenex Jupiter Proteo (250 × 4.60 mm) column (Phenomenex, Aschaffenburg, Germany) was used for analytical HPLC. The solvent system was A = H_2_O (0.1% TFA) and B = acetonitrile (0.1% TFA) with the following gradient: 0–1 min 5% B, 1–14 min 5–75% B, 14–16 min 75–95%, 16–18 min 95% B, 18–19 min 95 − 5% B, 19–20 min 5% B at a flow rate of 1 mL min^− 1^. Semi-preparative HPLC was performed on a Rigol HPLC system (Techlab, Braunschweig, Germany) in combination with a VDSpher 150 C18-E column (250 × 2.00 mm) (VDS optilab, Berlin, Germany) with the solvent system A = H_2_O (0.1% TFA) and B = acetonitrile (0.1% TFA). Samples were lyophilized using a Christ Alpha 1–2 LD plus lyophilizer (Christ, Oderode am Harz, Germany). Lyophilized peptides were stored at -20 °C. All instruments measuring radioactivity were calibrated and maintained in accordance with previously reported routine quality-control procedures^48^. Radioactivity was measured using an Activimeter ISOMED 2010 (Nuklear-Medizintechnik, Dresden, Germany).

### Peptides synthesis and chemical characterization of radiotracers

Custom made fluorescent peptides as well as f-MVPIKI were routinely synthesized with a purity of 95% by Synpeptides or Genscript. The peptide structures consist of six amino acids formylated at the N terminus. These peptides ligands were routinely dissolved in C1 buffer (130 mM NaCl, 10 mM HEPES, 5 mM KCl, 2 mM CaCl_2_, 5 mM glucose, pH 7.2) as a 1 mM stock solution. Strongly hydrophobic substances were dissolved as 30 mM stocks in DMSO (99.7%, Sigma-Aldrich). Stock solutions were routinely stored in small aliquots at -20 °C until use.

### Automated solid phase peptide synthesis (SPPS)

Peptides were synthesized with an automated ResPepSL synthesiser (Intavis Peptide Service, Tübingen, Germany) using Wang resin as the solid phase and Fmoc-protected amino acids (Carbolution, St. Ingbert, Germany) for coupling according to the method of Merrifield^49^. Formylation of the peptide as well as coupling of the spacer and the chelators was accomplished manually according to the literature. On-resin N-formylation: The in situ reagent *N*-formylimidazole was prepared by slow addition (drop wise over 15 min) of formic acid (60 mg, 49 µL; 1.3 mmol; in 2.95 mL dry DMF) to a suspension of N, N’-carbonyldiimidazole (115.3 mg; 1.2 mmol) in dry DMF (7 mL). The mixture was stirred for 1 h at room temperature prior to use. The solution (3.5 mL per 25 µmol peptide, 0.6 mmol) was applied to the resin and incubated for 18 h. Subsequently, remaining reagents were removed by washing the resin with DMF (30 mL) and DCM (30 mL).

### Manual SPPS

Removal of N-Alloc groups: The resin was soaked in DCM for 10 min prior to the deprotecting reaction. Dimethylamine borane (Me_2_NHBH_3_) (6 eq, 150 µmol, 8.84 mg) was dissolved in DCM (1 mL) and added to the resin under argon. Pd(PPh_3_)_4_ (0.1 eq, 25 µmol, 2.89 mg) was dissolved in DCM (1 mL) under argon and subsequently added to the resin-scavenger reaction tube. The reaction was stirred under argon for 10 min at RT. The resin was then rinsed with DCM (30 mL) and the procedure was repeated. The resin was then thoroughly rinsed with DCM (30 mL), DCM + 0.2% TFA (15 mL), DCM (30 mL), DCM + 5% DIPEA (30 mL) and DCM (30 mL).

Coupling of spacer and chelator: The resin was soaked in DMF for 10 min prior to the coupling reaction. HATU (4 eq, 100 µmol, 38 mg) and Ahx-Fmoc (4 eq, 100 µmol, 35 mg) or DOTA-tris-*t*Bu ester (4 eq, 100 µmol, 57 mg) were dissolved in DMF (1 mL, respectively). The solutions were added to the resin and the pH was adjusted with DIPEA (8 eq, 200 µmol, 25.8 mg, 35 µL) to pH 12. The coupling reaction was shaken for 150 min and every 30 min, the pH was checked and adjusted by the addition of DIPEA if necessary. Finally, the resin was thoroughly rinsed with DMF (30 mL) and DCM (30 mL).

Removal of Fmoc groups: The resin was soaked in DMF prior to the deprotection reaction followed by incubation in a 20% (v/v) piperidine in DMF solution (6 mL) for 30 min under shaking. The resin was then thoroughly rinsed with DMF (30 mL) and DCM (30 mL).

Cleavage of peptide conjugates: The resin-coupled peptides were cleaved and deprotected with a mixture of trifluoroacetic acid, ultrapure water and triisopropylsilane (ratio 95:2.5:2.5, v/v/v) followed by precipitation in cold diethyl ether. After centrifugation, the crude product was purified twice by semi-preparative HPLC. The first gradient was 0–20 min 5–95% B, 20–22 min 95% B, 22–23 min 95 − 5% B, 23–24 min 5% B at a flow rate of 7 mL/min. Fractions containing the product were unified and lyophilised. For the subsequent second purification, the following gradient was used: 0–20 min 40–60% B, 20–21 min 60–95% B, 21–23 min 95% B, 23–24 min 95 − 40% B, 24–25 min 40% B at a flow rate of 7 mL/min. Yield: 9.45 mg, 7.7 µmol, 31%; purity: 99%, HR-MS (MALDI-TOF): m/z calc. for [M]^+^ 1227.70172 found 1227.70570, analytical HPLC: *t*_R_ = 10.54 min.

^nat^Ga and ^nat^Lu complexes: Ga(NO_3_)_3_ (14.19 mg, 0.055 mmol) was dissolved in 0.2 M sodium acetate pH 4.0 (0.8 mL) and LuCl_3_ × 6 H_2_O (14.59 mg, 0.037 mmol) was dissolved in 0.1 M NH_4_Ac pH 5.4 (0.8 mL) and the peptide conjugate solution (200 µL; 1 nmol/mL) was added to each buffered metal salt solution. The solutions were incubated at 95 °C and 620 rpm for 30 min. After cooling to room temperature, the crude products were purified by semi preparative HPLC with the first gradient of the peptide purification. Fractions containing the product were unified and lyophilised.

^nat^Ga-f-MVPIK(Ahx-DOTA)I. Yield: 1.4 mg, 1.1 µmol, 73%; purity: 66%, HR-MS (MALDI-TOF): m/z calc. for [M + Ga]^+^ 1293.60382 found 1293.60161, analytical HPLC: t_R_ = 10.62 min.

^nat^Lu-f-MVPIK(Ahx-DOTA)I. Yield: 2.3 mg, 1.6 µmol, 80%; purity: 99%, HR-MS (MALDI-TOF): m/z calc. for [M + Lu]^+^ 1399.61902 found 1399.61544, analytical HPLC: t_R_ = 10.34 min.

Radiolabelling with ^68^Ga: [^68^Ga]GaCl_3_ was eluted from the generator indicated in the section of general methods and the activity was determined. A volume containing 25 MBq was taken for labelling. The total volume of the radiolabelling reaction was 200 µL. The buffer 0.2 M sodium acetate pH 4.0 (199 µL – V_activity_) was mixed with the peptide conjugate solution (1 µL, 1 nmol/µL) and 25 MBq of [^68^Ga]GaCl_3_. The reaction mixture was incubated at 95 °C for 15 min. Radiochemical yield was determined by analytical radio-HPLC. Radiochemical yield: 97%, radiochemical purity 69%, radio-HPLC: *t*_R_ = 11.03 min.

Radiolabelling with ^177^Lu: The peptide conjugate solution (1 µL, 1 nmol/µL) was added to the labelling buffer 0.1 M NH_4_Ac pH 5.4 (199 µL- V_activity_). A volume containing 170 MBq of [^177^Lu]LuCl_3_ was added to the labelling solution. The reaction mixture was incubated at 95 °C for 15 min. Radiochemical yield was determined by analytical radio-HPLC. Radiochemical yield 98%, radiochemical purity 92%, radio-HPLC: t_R_ = 10.95 min.

### Cell culture

The human brain glioblastoma cell line U87-MG, a malignant glioma, was obtained from CLS Cell Lines Service GmbH (300367) and HEK293T cells were purchased from American Type Culture Collection (ATCC). Cell culture was performed in a humidified incubator at 37 °C supplied with 5% CO_2_. Cells used for this study were not used for more than 25 passages. U87-MG cells were cultivated in modified Eagle’s medium (MEM-Biowest) supplemented with 10% FCS, 1% Glutamine, 1% non-essential amino acid and 1% penicillin/streptomycin. HEK293T cells were cultivated in Dulbecco’s modified Eagle’s medium (DMEM-Biowest) supplemented with 10% FCS, 1% Glutamine and 1% penicillin/streptomycin.

### Cultivation of HEK293T cells in 96-well plates

 Before seeding the HEK293T cells in 96-well plates, the wells were coated with poly-D-lysine (10 µg/mL in 1 × PBS), 50 µl of this solution were added to each well and incubated at RT for 20 min. After incubation, the solution was discarded, and 100 µL of cell suspension was pipetted into the wells and placed in a cell culture incubator at 37 °C and 5% CO_2_.

### Transient transfection of HEK293T cells

Cell transfection was performed approximately 24 h after seeding the cells, when they reached about 70% confluence. Transfection was carried out using jetPEI™ (Polyplus) according to the following protocol: 0.25 µg DNA was diluted in 10 µL NaCl (150 mM). 0.5 µL of jetPEI™ solution in 10 µL NaCl was mixed with the DNA solution and incubated at RT for 15–30 min. After incubation, 20 µL of the jetPEI™/DNA mixture was added dropwise to the cells, which were then placed in the cell culture incubator at 37 °C and 5% CO_2_. After 18 to 24 h of transfection, the medium was replaced, and 100 µL per well was added, then placed back in the incubator.

### Calcium imaging

Automated high-throughput Ca^2+^ measurements using a fluorescence imaging plate reader (FLIPR, Molecular Devices) were performed. Briefly, U87-MG cells were seeded in 96-well plates (~ 50,000 cells/well) one day before the experiments and incubated at 37 °C. HEK293T cells were co-transfected with the receptor or mock and the G protein Gα16 for 48 h. On the day of the experiments, the medium was removed, and the cells were loaded with a loading solution consisting of 2 µM Calbryte dye in C1 buffer (Calbryte 520AM, AAT Bioquest 20651) 50 µL per well, for at least 2 h at RT in the dark. After 2 h of loading with the fluorescent dye, the solution was removed, the plate was washed three times using an ELX50 washer (BioTek), and the intracellular release of calcium was measured using FLIPR. A Ca^2+^ response was defined as an increase in the excitation ratio (340 nm/380 nm) that was at least four times higher than the mean baseline noise after bath application.

### Ligand binding analysis of FPR-HEK293T

To characterize the binding capability of the peptide to its specific receptor in HEK293T cells transiently transfected with FPR1, FPR2, FPR3, or mock (empty vector as negative control) for 24 h. The affinity and stability of peptide and modified peptide probes were measured on the cells. Briefly, transfected HEK293T cells were cultured overnight at 37 °C, followed by the addition of a fluorescent ligand dilution series for different time points. Rinsed and washed three times with C1 buffer. The binding was analyzed using confocal microscopy. FITC or TAMRA fluorescence visualize the binding of the f-MVPIK(FITC)I or f-MVPIK(TAMRA)I peptide to the FPR receptor, respectively. Hoechst fluorescence detects cell nuclei. The experiments were performed three times in triplicate.

Binding affinity: To examine the binding affinity of f-MVPIK(FITC)I to the FPR1 receptor, a time-dependent binding affinity was analyzed at different time points. HEK293T cells were transiently transfected with FPR1 or mock as a negative control. After 24 h post-transfection, the medium was removed, and cells were stimulated with 1 µM f-MVPIK(FITC)I in DMEM medium at 37 °C for specific time points (1, 5, and 30 min). After incubation, cells were rapidly washed three times with C1 buffer, and fluorescence intensity was measured over time by confocal microscopy.

Stability of receptor-ligand binding: HEK293T cells were transiently transfected with FPR1 or mock as a negative control. 24 h after transfection, the medium was removed, and cells were stimulated for 30 min at 37 °C with 1 µM f-MVPIK(FITC)I ligand. Cells were washed once with DMEM medium to remove unbound ligand. The plate was placed on ice for 2 min, and cells were then permeabilized with 0.1% Triton-X 100 in DMEM with Hoechst nuclear staining, incubated for 20 min at RT. The cells were washed three times with C1 buffer to remove excess Triton-X 100 and Hoechst stain. Fluorescence images were captured and analyzed using confocal microscopy.

Stability of ligand uptake: The stability of cellular uptake of the peptide f-MVPIK(FITC)I was investigated as follows: HEK293T cells were transiently transfected with FPR1 or mock as a negative control for 24 h. After 24 h of transfection, the medium was removed, and cells were stimulated with 1 µM f-MVPIK(FITC)I in DMEM medium at 37 °C for 30 min. After incubation, the cells were washed three times with C1 buffer, and the loss of fluorescence intensity was observed by confocal microscopy at specific time points (24, 48, and 72 h).

Receptor-mediated endocytosis of fluorescent ligands: To measure receptor-mediated endocytosis of fluorescent ligands, HEK293T cells were transiently transfected with FPR1, FPR2, FPR3, or mock for 24 h and incubated for 60 min at 37 °C with a concentration range from 3 µM to 1 nM of fluorescent ligand f-MVPIK(FITC)I. At the end of this time, nuclear staining was performed using Hoechst in medium for 30 min at 37 °C. After the incubation period, the plates were washed three times with C1 buffer, and fluorescence intensity was recorded and analyzed using a confocal microscope.

### Ligand binding analysis of FPR1 expressing U87-MG

U87-MG cells were seeded at 10^5^ cells/well in a 96-well plate, incubated for 24 h at 37 °C, followed by the addition of the fluorescent ligand and incubation for 2 h at 37 °C. Quantification of cell-associated fluorescence was assessed using confocal microscopy. FITC fluorescence visualized the binding of the f-MVPIK(FITC)I peptide to the FPR1 receptor, and Hoechst fluorescence detected cell nuclei. The experiments were performed three times in triplicate.

### RNA isolation and quantitative polymerase chain reaction (RT-qPCR)

The measurement of mRNA expression levels of FPR1 was performed using RT-qPCR. Absolute quantification of the signals was carried out by normalizing the target genes to the housekeeping reference gene glyceraldehyde-3-phosphate dehydrogenase (GAPDH) and calculated according to specific standard curves, as previously described^38^. The qPCR products were electrophoresed on a 1.5% agarose gel and visualized with Roti-Gelstain (Carl Roth) incorporation under UV light.

### In vitro 3D spheroid generation

We used three-dimensional (3D) in vitro models as an intermediate model between in vitro cancer cell line cultures and in vivo tumors. First, the optimal seeding density for tumor spheroid formation was determined by examining the cell seeding density of U87-MG cell lines (Supplement. 3). For spheroid generation, 200 µL/well of U87-MG cell suspensions at an optimized density of 2,000 cells/well were dispensed into 96-well round-bottom plates (Brand Plates-Pure Grade TMs) mounted with 1 mg/ml growth factor collagen Typ IV (Thermo Fischer). Plates were incubated for 4 days at 37 °C, 5% CO2, and 95% humidity in an IncuCyte S3 (Essen Bioscience) to tracking the spheroid formation and growth. In this work, we tested the binding and internalization of f-MVPIK(FITC)I in the 3D tumor spheroids. Images were acquired and analyzed using ImageXpress Micro confocal microscopy.

### Stability in cell culture medium

The radiolabelled compound (50 µL) was added to complete growth medium (MEM + 10% FBS, 500 µL) and incubated at 37 °C. Samples of 100 µL were taken at selected time points. The plasma proteins were precipitated with ice-cold acetonitrile and the mixture was centrifuged for 1 min at 1500 rpm. The supernatant was analyzed by analytical radio-HPLC.

### Stability in human plasma

The radiolabelled compound (50 µL) was added to male human AB plasma (500 µL, Sigma Aldrich, USA, male AB) and incubated at 37 °C. Samples of 100 µL were taken at selected time points. The plasma proteins were precipitated with acetonitrile (-20 °C) and the precipitate was centrifuged for 1 min at 1500 rpm. The supernatant was analyzed by analytical radio-HPLC.

### Cell internalization

The medium was removed from the wells and replaced by fresh complete growth medium (1.3 mL per well). The inhibitor solution BOC-FLFLF (1000-fold excess against the radiotracer, in PBS/DMSO) was added to the wells in the bottom row (100 µL per well). A solution of PBS and DMSO in the same ratio as the inhibitor solution was added to the wells of the top row (100 µL per well). To each well, the radiotracer solution in the desired concentration was added (100 µL per well). The plate was incubated at 37 °C for the time specified for each experiment. The supernatant was removed, and the cells were placed on ice and rinsed twice with ice cold PBS (1 mL per well). Sodium hydroxide (1 M, 2 × 1 mL) was added to detach the cells and the suspension was collected in counting tubes. The tubes were analyzed with an HIDEX gamma counter (HIDEX, Turku, Finland) using the appropriate detection window for the corresponding radionuclide.

### Ex vivo biodistribution and planar scintigraphy imaging studies

All experiments involving animals were performed in accordance with an animal experimentation license approved by the Zurich Cantonal Veterinary Office, Switzerland (Jason P. Holland; license number ZH095/2023), and in compliance with the German Animal Welfare Act. This study is reported in accordance with the ARRIVE guidelines (https://arriveguidelines.org). Six to eight-week old male NOD/SCID mice (17–20 g) were obtained from Charles River Laboratories (Lyon, France). Mice were provided with food and water *ad libitum*. For planar scintigraphy imaging and ex vivo biodistribution studies, mice were injected with 100 µL sterile saline formulations of ~ 250 pmol (~ 10 MBq) [^177^Lu]Lu-f-MVPIK(Ahx-DOTA)I by intravenous tail-vein injection and anesthetized with isoflurane (2–4% in air). Imaging was performed on Gamma-eye scanner (Bioemtech, Greece) at 5 min and 1 h p.i. Directly after imaging at 1 h p.i., animals were euthanised by isoflurane asphyxiation followed by terminal exsanguination. A total of 13 tissues were removed, rinsed in water, dried in air for approx. 2 min., weighed and counted on a calibrated gamma counter for accumulation of activity. The mass of radiotracer formulation injected into each animal was measured and used to determine the total number of counts per minute (cpm) injected into each mouse by comparison to a standard syringe of known activity and mass. Count data were background- and decay-corrected, and the tissue uptake for each sample (determined in units of percentage injected dose per gram [%ID g^− 1^]) was calculated by normalization to the total amount of activity injected for each individual animal.

### Statistical analysis

 The results are expressed as the means ± standard deviations of the number of the indicated determinations. Calcium measurement experiments were routinely performed as triplicates and averaged over at least three independent transfections. Response amplitudes (F/F_0_) were calculated by dividing maximal fluorescence change after ligand application by baseline fluorescence. Maximal amplitudes were set to 100%, and curves were calculated with Graph Pad Prism Version 10.1 (GraphPad Software, Boston, MA, USA). S.D. values were calculated as averages from independently obtained EC_50_ values using the STDEV. Data derived from experiments were analyzed by two-way ANOVA. *P* < 0.05 was accepted as significant.

## Supplementary Information

Below is the link to the electronic supplementary material.

Supplementary Material 1

Supplementary Material 2

## Data Availability

All data generated and/or analyzed as part of the current study are included in this article and its supplementary data as well as in the original supplementary files.

## References

[1] Bufe, B. et al. Recognition of bacterial signal peptides by mammalian formyl peptide receptors. *J. Biol. Chem.* **290**(12), 7369–7387. 10.1074/jbc.M114.626747 (2015). 25605714 10.1074/jbc.M114.626747 PMC4367248

[2] Bufe, B., Schumann, T. & Zufall, F. Formyl peptide receptors from immune and vomeronasal system exhibit distinct agonist properties. *J. Biol. Chem.* **287**(40), 33644–33655. 10.1074/jbc.M112.375774 (2012). 22859307 10.1074/jbc.M112.375774 PMC3460462

[3] He, H. Q. & Ye, R. The formyl peptide receptors: diversity of ligands and mechanism for recognition. *Molecules* **22**(3), 455. 10.3390/molecules22030455 (2017). 28335409 10.3390/molecules22030455 PMC6155412

[4] Ye, R. D. et al. International union of basic and clinical pharmacology. LXXIII. Nomenclature for the formyl peptide receptor (FPR) family. *Pharmacol. Rev.* **61**(2), 119–161. 10.1124/pr.109.001578 (2009). 19498085 10.1124/pr.109.001578 PMC2745437

[5] Bloes, D. A., Kretschmer, D. & Peschel, A. Enemy attraction: bacterial agonists for leukocyte chemotaxis receptors. *Nat. Rev. Microbiol.* **13**(2), 95–104. 10.1038/nrmicro3390 (2015). 25534805 10.1038/nrmicro3390

[6] Krepel, S. A. & Wang, J. M. Chemotactic ligands that activate G-protein-coupled formylpeptide receptors. *Int. J. Mol. Sci.* **20**(14), 3426. 10.3390/ijms20143426 (2019). 31336833 10.3390/ijms20143426 PMC6678346

[7] Li, L. et al. New development in studies of formyl-peptide receptors: critical roles in host defense. *J. Leukoc. Biol.* **99**(3), 425–435. 10.1189/jlb.2RI0815-354RR (2016). 26701131 10.1189/jlb.2RI0815-354RR PMC4750370

[8] Migeotte, I., Communi, D. & Parmentier, M. Formyl peptide receptors: A promiscuous subfamily of G protein-coupled receptors controlling immune responses. *Cytokine Growth Factor Rev.* **17**(6), 501–519. 10.1016/j.cytogfr.2006.09.009 (2006). 17084101 10.1016/j.cytogfr.2006.09.009

[9] Li, Y. & Ye, D. Molecular biology for formyl peptide receptors in human diseases. *J. Mol. Med.* **91**(7), 781–789. 10.1007/s00109-013-1005-5 (2013). 23404331 10.1007/s00109-013-1005-5

[10] Van Der Vorst, E. P. C. et al. G-Protein coupled receptor targeting on myeloid cells in atherosclerosis. *Front. Pharmacol.* **10**, 531. 10.3389/fphar.2019.00531 (2019). 31191301 10.3389/fphar.2019.00531 PMC6540917

[11] Prevete, N., Liotti, F., Marone, G., Melillo, R. M. & De Paulis, A. Formyl peptide receptors at the interface of inflammation, angiogenesis and tumor growth. *Pharmacol. Res.* **102**, 184–191. 10.1016/j.phrs.2015.09.017 (2015). 26466865 10.1016/j.phrs.2015.09.017

[12] Rongvaux, A. Innate immunity and tolerance toward mitochondria. *Mitochondrion* **41**, 14–20. 10.1016/j.mito.2017.10.007 (2018). 29054471 10.1016/j.mito.2017.10.007

[13] Ong, W. Y. & Chua, J. E. Role of formyl peptide receptor 2 (FPR2) in the normal brain and in neurological conditions. *Neural Regeneration Res.* **14**(12), 2071. 10.4103/1673-5374.262575 (2019). 10.4103/1673-5374.262575 PMC6788234 31397336

[14] Zhou, Y. et al. Formylpeptide receptor FPR and the rapid growth of malignant human gliomas. *JNCI: J. Natl. Cancer Inst.* **97**(11), 823–835. 10.1093/jnci/dji142 (2005). 15928303 10.1093/jnci/dji142

[15] Minopoli, M. et al. Targeting the formyl peptide receptor type 1 to prevent the adhesion of ovarian cancer cells onto mesothelium and subsequent invasion. *J. Experimental Clin. Cancer Res.* **38**(1), 459. 10.1186/s13046-019-1465-8 (2019). 10.1186/s13046-019-1465-8 PMC6839174 31703596

[16] Prevete, N. et al. The formyl peptide receptor 1 exerts a tumor suppressor function in human gastric cancer by inhibiting angiogenesis. *Oncogene* **34**(29), 3826–3838. 10.1038/onc.2014.309 (2015). 25263443 10.1038/onc.2014.309

[17] Jiang, X., Lei, T. & Zhang, M. Expression and functions of formyl peptide receptor 1 in drug-resistant bladder cancer. *Technol. Cancer Res. Treat.* **17**, 1533034618769413. 10.1177/1533034618769413 (2018). 29665744 10.1177/1533034618769413 PMC5912276

[18] Boer, J. C. et al. Microenvironment involved in FPR1 expression by human glioblastomas. *J. Neurooncol.* **123**(1), 53–63. 10.1007/s11060-015-1777-2 (2015a). 25894595 10.1007/s11060-015-1777-2 PMC4439437

[19] Bastien, J. I. L., McNeill, K. A. & Fine, H. A. Molecular characterizations of glioblastoma, targeted therapy, and clinical results to date. *Cancer* **121**(4), 502–516. 10.1002/cncr.28968 (2015). 25250735 10.1002/cncr.28968

[20] Morris, S. et al. Whole blood FPR1 mRNA expression predicts both non-small cell and small cell lung cancer. *Int. J. Cancer*. **142**(11), 2355–2362. 10.1002/ijc.31245 (2018). 29313979 10.1002/ijc.31245 PMC5901395

[21] Zhu, J. et al. The role of formyl peptide receptors in neurological diseases via regulating inflammation. *Front. Cell. Neurosci.* **15**, 753832. 10.3389/fncel.2021.753832 (2021a). 34650406 10.3389/fncel.2021.753832 PMC8510628

[22] Li, S. Q. et al. The expression of formyl peptide receptor 1 is correlated with tumor invasion of human colorectal cancer. *Sci. Rep.* **7**(1), 5918. 10.1038/s41598-017-06368-9 (2017). 28724995 10.1038/s41598-017-06368-9 PMC5517416

[23] Prevete, N. et al. Formyl peptide receptor 1 suppresses gastric cancer angiogenesis and growth by exploiting inflammation resolution pathways. *OncoImmunology* **6**(4), e1293213. 10.1080/2162402X.2017.1293213 (2017). 28507800 10.1080/2162402X.2017.1293213 PMC5414878

[24] Kwekkeboom, D., Krenning, E. P. & de Jong, M. Peptide receptor imaging and therapy. *J. Nuclear Medicine: Official Publication Soc. Nuclear Med.* **41**(10), 1704–1713 (2000). 11038002

[25] Nock, B. A., Kanellopoulos, P., Joosten, L., Mansi, R. & Maina, T. Peptide radioligands in cancer theranostics: agonists and antagonists. *Pharmaceuticals* **16**(5), 674. 10.3390/ph16050674 (2023). 37242457 10.3390/ph16050674 PMC10222684

[26] Pellico, J. et al. In vivo imaging of lung inflammation with neutrophil-specific 68Ga nano-radiotracer. *Sci. Rep.* **7**(1), 13242. 10.1038/s41598-017-12829-y (2017). 29038592 10.1038/s41598-017-12829-y PMC5643527

[27] Mattila, J. T. et al. Retention of 64Cu-FLFLF, a formyl peptide receptor 1-Specific PET probe, correlates with macrophage and neutrophil abundance in lung granulomas from cynomolgus macaques. *ACS Infect. Dis.* **7**(8), 2264–2276. 10.1021/acsinfecdis.0c00826 (2021). 34255474 10.1021/acsinfecdis.0c00826 PMC8744071

[28] Yang, X. et al. Targeting formyl peptide receptor 1 of activated macrophages to monitor inflammation of experimental osteoarthritis in rat. *J. Orthop. Research: Official Publication Orthop. Res. Soc.* **34**(9), 1529–1538. 10.1002/jor.23148 (2016). 10.1002/jor.23148 26717557

[29] Locke, L. W. et al. A novel neutrophil-specific PET imaging agent: cFLFLFK-PEG-64Cu. *J. Nuclear Medicine: Official Publication Soc. Nuclear Med.* **50**(5), 790–797. 10.2967/jnumed.108.056127 (2009). 10.2967/jnumed.108.056127 PMC3004780 19372473

[30] Zhang, Y. et al. Neutrophil targeting heterobivalent SPECT imaging probe: cFLFLF-PEG-TKPPR-99mTc. *Bioconjug. Chem.* **21**(10), 1788–1793. 10.1021/bc100063a (2010). 20843030 10.1021/bc100063a

[31] Arbyn, M. et al. Estimates of incidence and mortality of cervical cancer in 2018: A worldwide analysis. *Lancet Global Health*. **8**(2), e191–e203. 10.1016/S2214-109X(19)30482-6 (2020). 31812369 10.1016/S2214-109X(19)30482-6 PMC7025157

[32] Cramer, S. W. & Chen, C. C. Photodynamic therapy for the treatment of glioblastoma. *Front. Surg.* **6**, 81. 10.3389/fsurg.2019.00081 (2019). 32039232 10.3389/fsurg.2019.00081 PMC6985206

[33] Novy, Z. et al. Preclinical evaluation of radiolabeled peptides for PET imaging of glioblastoma multiforme. *Molecules* **24**(13), 2496. 10.3390/molecules24132496 (2019). 31288488 10.3390/molecules24132496 PMC6651196

[34] Lind, S., Dahlgren, C., Holmdahl, R., Olofsson, P. & Forsman, H. Functional selective FPR1 signaling in favor of an activation of the neutrophil superoxide generating NOX2 complex. *J. Leukoc. Biol.* **109**(6), 1105–1120. 10.1002/JLB.2HI0520-317R (2021). 33040403 10.1002/JLB.2HI0520-317R PMC8246850

[35] Nafiz, T. N. et al. Differential requirement of formyl peptide receptor 1 in macrophages and neutrophils in the host defense against Mycobacterium tuberculosis infection. *Res. Sq.*, rs.3.rs-4421561. 10.21203/rs.3.rs-4421561/v1 (2024). 10.1038/s41598-024-71180-1 PMC11464745 39384825

[36] Strouse, J. J. et al. A novel fluorescent cross-reactive formylpeptide receptor/formylpeptide receptor-like 1 hexapeptide ligand. *Cytometry Part. A: J. Int. Soc. Anal. Cytol.* **75**(3), 264–270. 10.1002/cyto.a.20670 (2009a). 10.1002/cyto.a.20670 PMC2917377 19006074

[37] Wang, J., Chen, M., Li, S. & Ye, R. D. Targeted delivery of a ligand–drug conjugate via formyl peptide receptor 1 through cholesterol-dependent endocytosis. *Mol. Pharm.* **16**(6), 2636–2647. 10.1021/acs.molpharmaceut.9b00188 (2019). 31067065 10.1021/acs.molpharmaceut.9b00188

[38] Ahmet, D. S. et al. Application of small molecule FPR1 antagonists in the treatment of cancers. *Sci. Rep.* **10**(1), 17249. 10.1038/s41598-020-74350-z (2020). 33057069 10.1038/s41598-020-74350-z PMC7560711

[39] Busch, L. et al. Amyloid beta and its naturally occurring N-terminal variants are potent activators of human and mouse formyl peptide receptor 1. *J. Biol. Chem.* **298**(12), 102642. 10.1016/j.jbc.2022.102642 (2022a). 36309087 10.1016/j.jbc.2022.102642 PMC9694488

[40] Yao, X. et al. Chemoattractant receptors as Pharmacological targets for elimination of glioma stem-like cells. *Int. Immunopharmacol.* **11**(12), 1961–1966. 10.1016/j.intimp.2011.08.021 (2011). 21930249 10.1016/j.intimp.2011.08.021 PMC3224200

[41] Dufton, N. & Perretti, M. Therapeutic anti-inflammatory potential of formyl-peptide receptor agonists. *Pharmacol. Ther.* **127**(2), 175–188. 10.1016/j.pharmthera.2010.04.010 (2010). 20546777 10.1016/j.pharmthera.2010.04.010

[42] Yao, X. H. et al. Production of angiogenic factors by human glioblastoma cells following activation of the G-protein coupled formylpeptide receptor FPR. *J. Neurooncol.* **86**(1), 47–53. 10.1007/s11060-007-9443-y (2008). 17611713 10.1007/s11060-007-9443-y

[43] Gallo, I. et al. Formyl peptide receptor as a novel therapeutic target for anxiety-related disorders. *PloS One*. **9**(12), e114626. 10.1371/journal.pone.0114626 (2014). 25517119 10.1371/journal.pone.0114626 PMC4269406

[44] He, H. Q. et al. Functional characterization of three mouse formyl peptide receptors. *Mol. Pharmacol.* **83**(2), 389–398. 10.1124/mol.112.081315 (2013). 23160941 10.1124/mol.112.081315 PMC4170117

[45] Raabe, C. A., Gröper, J. & Rescher, U. Biased perspectives on formyl peptide receptors. *Biochim. Et Biophys. Acta (BBA) - Mol. Cell. Res.* **1866**(2), 305–316. 10.1016/j.bbamcr.2018.11.015 (2019). 10.1016/j.bbamcr.2018.11.015 30521870

[46] Liu, M. et al. G protein-coupled receptor FPR1 as a pharmacologic target in inflammation and human glioblastoma. *Int. Immunopharmacol.* **14**(3), 283–288. 10.1016/j.intimp.2012.07.015 (2012). 22863814 10.1016/j.intimp.2012.07.015 PMC3547636

[47] Xu, X., Farach-Carson, M. C. & Jia, X. Three-dimensional in vitro tumor models for cancer research and drug evaluation. *Biotechnol. Adv.* **32**(7), 1256–1268. 10.1016/j.biotechadv.2014.07.009 (2014). 25116894 10.1016/j.biotechadv.2014.07.009 PMC4171250

[48] Zanzonico, P. et al. Routine quality control of clinical nuclear medicine instrumentation: A brief review. *J. Nuclear Medicine: Official Publication Soc. Nuclear Med.* **49**(7), 1114–1131. 10.2967/jnumed.107.050203 (2008). 10.2967/jnumed.107.050203 PMC2703015 18587088

[49] Merrifield, R. B. Solid phase peptide synthesis. I. The synthesis of a tetrapeptide. *J. Am. Chem. Soc.* **85**(14), 2149–2154. 10.1021/ja00897a025 (1963).

