# Correction: Assessing the potential of bacterial signal peptides for radiopharmaceutical applications

**DOI:** 10.1038/s41598-026-36486-2

**Published:** 2026-02-03

**Authors:** Zukaa Al Taleb, Ina Hierlmeier, Heiko Heilmann, Martin Jung, Mark Bartholomä, Bernd Bufe

**Affiliations:** 1 Department of Informatics and Microsystems Technology, University of Applied Sciences Kaiserslautern, 66482 Zweibrücken, Germany; 2 Department of Nuclear Medicine, Medical Center, PharmaScienceHub (PSH), Saarland University, 66421 Homburg, Germany; 3 Medical Biochemistry and Molecular Biology, Saarland University, 66421 Homburg, Germany

Correction to: *Scientific Reports* 10.1038/s41598-025-18831-z, published online 12 September 2025

In the original version of this Article Mark Bartholomä and Bernd Bufe were omitted as equally contributing authors.

In addition, affiliation 2 contained an error which was incorrectly given as ‘Department of Nuclear Medicine, Medical Center, Saarland University, 66,421 Homburg, Germany.’ The correct affiliation is listed here: Department of Nuclear Medicine, Medical Center, PharmaScienceHub (PSH), Saarland University, 66,421 Homburg, Germany.

Finally, the Author Contributions section,

“B.B, M.B and Z.A wrote the main manuscript text . H.H prepared figures 1(a-c) 2(c) . Z.A prepared figures 1(b-d),2(a-b-d),3(b-c),4(a-b-c-d-e). I.H and M.J prepared figure 3(a), 5(a-b-c-d) and M.B prepared figure 6 (a-b-c-d-e).”

now reads:

“B.B, M.B, I.H. and Z.A. planned and designed the experiments and interpreted the results. Z.A. performed the calcium imaging, binding study and RT-qPCR experiments and supplements. H.H. performed and analyzed the binding study, calcium imaging and endocytosis experiments. I.H and M.J performed the peptide synthesis. I.H. performed the radiolabelings, HPLC measurements, and cell assays with radioactive probes. M.B. performed the planar scintigraphy imaging and ex vivo biodistribution studies. Manuscript was written by Z.A. and all authors reviewed the manuscript.”

The original Article has been corrected.

