# Supplementary material for: Assessing the potential of bacterial signal peptides for radiopharmaceutical applications
Source: Sci Rep. 2025 Sep 12;15:32479. doi: 10.1038/s41598-025-18831-z (PMC12432127; doi:10.1038/s41598-025-18831-z)
Supplement: Supplementary file 1 — Supplementary Material 1 [file 41598_2025_18831_MOESM1_ESM.pdf]

**Supporting Fig 1, 2 and 3:**

**Analysis and quantification of fluorescence signals intensity using Confocal microscopy technique:**

**Changes in intensity over time or under different conditions were quantified as explained:**

The FITC and DAPI filter sets are used for fluorescent ligands with the FITC fluorescent labeling. Another filter set is the Texas Red filter. This is used for ligands with the TAMRA fluorescent label. The exposure times are 200 ms for FITC and TAMRA (ligands) and 50 ms for DAPI (nuclear staining). In each case, 2 sites per well are recorded. Various module sets (Table1) are used to evaluate the binding study, which process the measurement parameters and output them at the end.

After processing the measurement parameters with the modules in Table 1, the total cell count and the sums of the fluorescence intensities are output. The intensity per cell can be calculated from the fluorescence intensity sums and the total cell count (Example 1)

Tabel 1: **Modules for evaluating the binding study:**

| **Module** | **Parameters** | **Module setting** | **Module group** |
|---|---|---|---|
| All cores | Filter Min. width<br>Max. Width<br>Intensity over background | DAPI (5-15µm)<br>250 a.u. | Cell Scoring Module |
| Positive Marker | Filter<br>Gefärbte Flächen<br>Min. Weite<br>Max. Weite<br>Intensität über Hintergrund<br>Algorithmus Typ | FITC oder TAMRA<br>Beide<br>7 µm<br>20 µm<br>120 a.u. | Cell Scoring Module |
| Total intensity | Ligand mask<br>filter<br>Min. width<br>Max. Width<br>Intensity over background | FITC or TAMRA<br>0 µm<br>30 µm<br>150 a.u. | |
| Total cell count | Nuclear mask<br>filter<br>Min. width<br>Max. width<br>Intensity over background | DAPI<br>5 µm<br>15 µm<br>250 a.u. | |
| | | | |
| | | | |

**Example1: Modules for evaluating the binding study:**

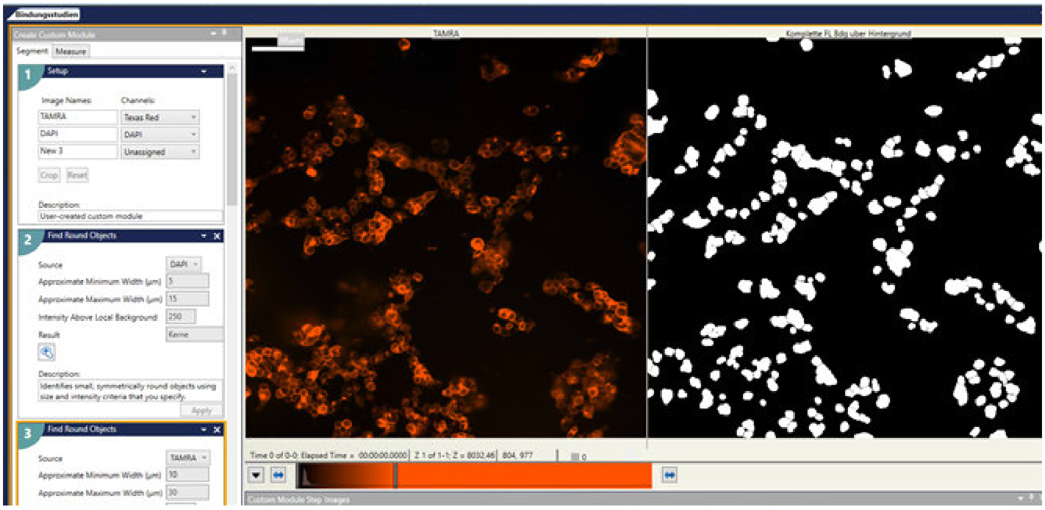


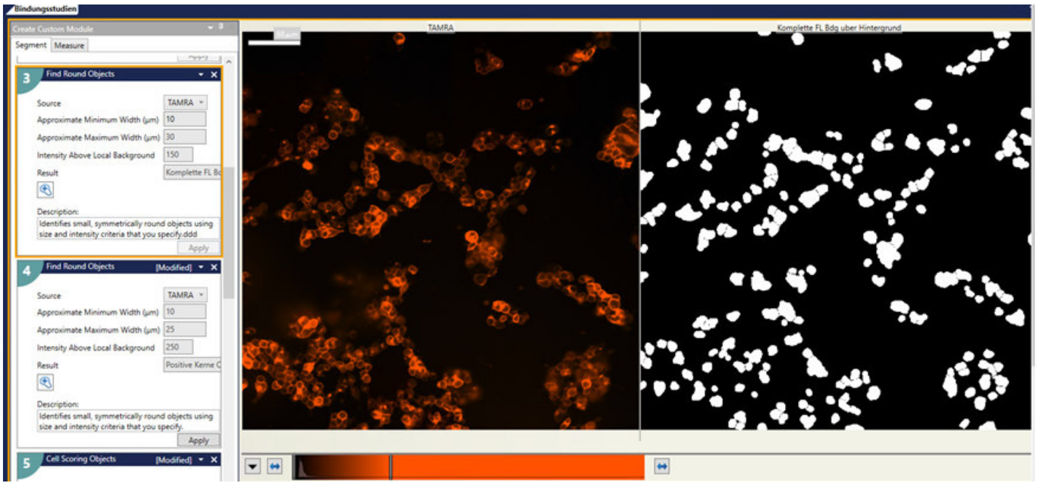


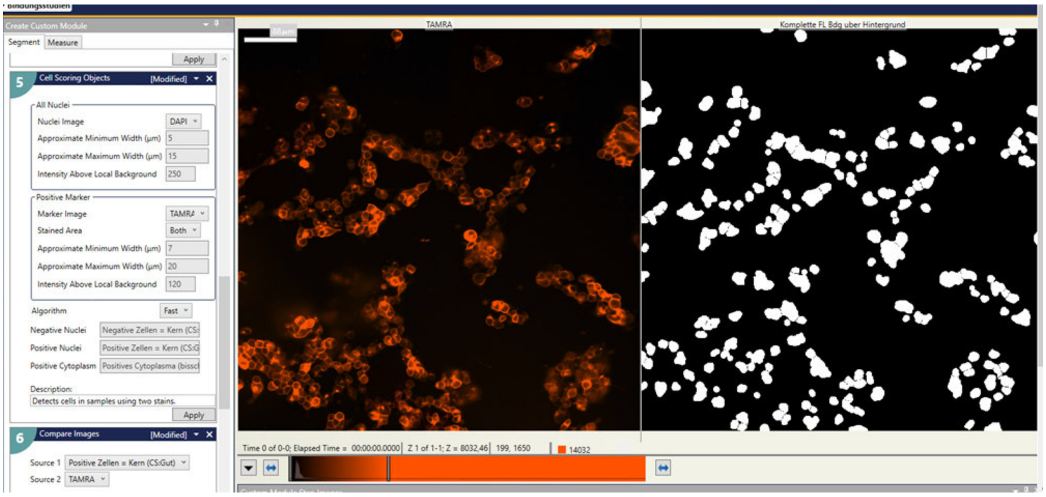

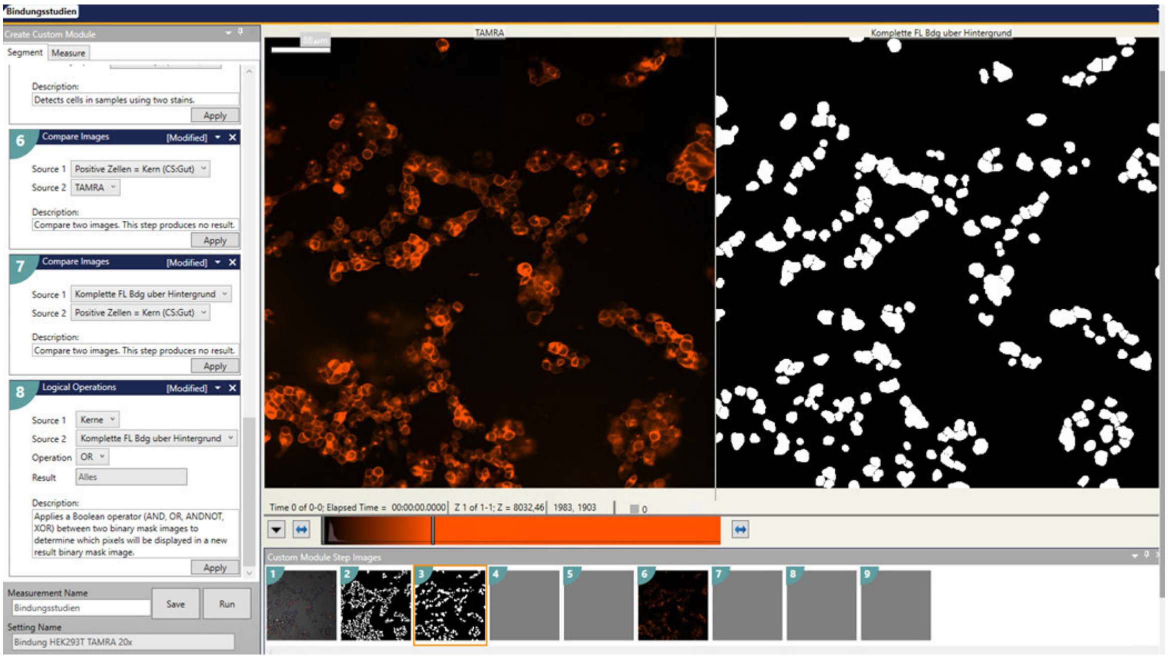
Bindungsstudien
Create Custom Module
Segment
Measure
Description:
Detects cells in samples using two stains.
Apply
6
Compare Images
[Modified]
Source 1
Positive Zellen = Kern (CS:Gut)
Source 2
TAMRA
Description:
Compare two images. This step produces no result.
Apply
7
Compare Images
[Modified]
Source 1
Komplette FL Bdg uber Hintergrund
Source 2
Positive Zellen = Kern (CS:Gut)
Description:
Compare two images. This step produces no result.
Apply
8
Logical Operations
[Modified]
Source 1
Kerne
Source 2
Komplette FL Bdg uber Hintergrund
Operation
OR
Result
Alles
Description:
Applies a Boolean operator (AND, OR, ANDNOT, XOR) between two binary mask images to determine which pixels will be displayed in a new result binary mask image.
Apply
Measurement Name
Bindungsstudien
Save
Run
Setting Name
Bindung HEK293T TAMRA 20x
TAMRA
Komplette FL Bdg uber Hintergrund
Time 0 of 0-0; Elapsed Time = 00:00:00.0000
Z 1 of 1-1; Z = 8032,46
1983, 1903
0
Custom Module Step Images
1
2
3
4
5
6
7
8
9

Supporting Fig 1c

Binding of f-MVPIK(FITC)I to FPR in HEK 293T

| centration log | | | | | | Mock | | | | | | | | | |
|---|---|---|---|---|---|---|---|---|---|---|---|---|---|---|---|
| -5,5 | 0,00285723 | 0,00699217 | 0,00983944 | 0,00639502 | 0,00914195 | 0,00365687 | 0,00354058 | 0,00121646 | 0,00230086 | 0,00431703 | 0,00510969 | 0 | 0,00557121 | 0,00104308 | 0,01673065 |
| -6 | 0,00253686 | 0,00107848 | 0 | 0,00093752 | 0,00426266 | 0 | 0,00098689 | 0,00224252 | 0,00602925 | 0 | 0,00123038 | 0,00102906 | 0,00097452 | 0,00119085 | 0 |
| -6,5 | 0 | 0,00121329 | 0,00142641 | 0 | 0,00589467 | 0 | 0,00092131 | 0,00199633 | 0,002423 | 0,0009046 | 0 | 0,00255998 | 0 | 0,00086336 | 0,00080605 |
| -7 | 0 | 0 | 0 | 0 | 0,01052367 | 0 | 0,00192847 | 0,00947174 | 0 | 0,00093725 | 0,00102596 | 0 | 0 | 0,00103632 | 0,00128952 |
| -7,5 | 0 | 0 | 0,002829 | 0 | 0 | 0,0012825 | 0,00248642 | 0,00115882 | 0,00835654 | 0 | 0,00143657 | 0 | 0 | 0,0014772 | 0 |
| -8 | 0 | 0 | 0,00517689 | 0,00393797 | 0 | 0 | 0,00665234 | 0 | 0,01116496 | 0,00097539 | 0,00310477 | 0 | 0,00093494 | 0,0020626 | 0,00209637 |
| -8,5 | 0 | 0,0014725 | 0 | 0 | 0 | 0,00279169 | 0,001695 | 0 | 0 | 0 | 0 | 0,00345622 | 0,00885044 | 0,00256068 | 0,00151567 |
| -9 | 0,00140238 | 0 | 0 | 0,00275153 | 0 | 0,00394856 | 0 | 0 | 0 | 0 | 0,00834473 | 0,00465958 | 0 | 0,00962544 | 0 |
| -9,5 | 0 | 0,00420247 | 0 | 0,00095354 | 0,001955 | 0 | 0 | 0 | 0 | 0,00094292 | 0,00098935 | 0,00170786 | 0,00441664 | 0,00208277 | 0 |
| -10 | 0 | 0,00145165 | 0 | 0,00160533 | 0 | 0,00815085 | 0,00101207 | 0 | 0,00132757 | 0 | 0,00522861 | 0,00235099 | 0,00151753 | 0,00507861 | 0,00100616 |

| centration log | | | | | | FPR1 | | | | | | | | | |
|---|---|---|---|---|---|---|---|---|---|---|---|---|---|---|---|
| -5,5 | 1 | 0,9425633 | 0,95820578 | 0,96961086 | 0,86705995 | 1 | 0,98002405 | 0,91842356 | 0,99563354 | 0,88296851 | 0,9059113 | 0,66202583 | 0,96402623 | 1 | 0,8256064 |
| -6 | 0,94598533 | 1 | 0,85716594 | 1 | 1 | 0,89409131 | 0,97725772 | 1 | 0,86991524 | 1 | 1 | 0,87518997 | 0,84774056 | 0,98974618 | 0,86570815 |
| -6,5 | 0,93820566 | 0,81499927 | 0,99760984 | 0,87511486 | 0,91344818 | 0,84939366 | 1 | 0,96783036 | 0,86045335 | 0,98979297 | 0,9850919 | 1 | 1 | 0,97346491 | 1 |
| -7 | 0,93038341 | 0,9685129 | 0,96703469 | 0,79044101 | 0,88711225 | 0,89756155 | 0,82164775 | 0,94657575 | 0,97399254 | 0,92215081 | 0,91342248 | 0,84532967 | 0,90984738 | 0,98420569 | 0,96965258 |
| -7,5 | 0,96408248 | 0,90743551 | 0,91996207 | 0,70647065 | 0,78642383 | 0,79040495 | 0,93457106 | 0,84139796 | 0,7731698 | 0,92430797 | 0,85797139 | 0,76124003 | 0,94524918 | 0,87694064 | 0,82879636 |
| -8 | 0,89624892 | 0,93345532 | 1 | 0,65566298 | 0,58682361 | 0,67435194 | 0,78630442 | 0,62769644 | 0,82701468 | 0,74792627 | 0,96747495 | 0,72259953 | 0,87732633 | 0,90846643 | 0,79526179 |
| -8,5 | 0,85268127 | 0,78965422 | 0,72096257 | 0,62409131 | 0,58731033 | 0,50815 | 0,64081788 | 0,7292513 | 1 | 0,8147345 | 0,81296437 | 0,80449571 | 0,84896392 | 0,8053427 | 0,85304591 |
| -9 | 0,29388646 | 0,31704518 | 0,20017729 | 0,28977596 | 0,26243815 | 0,12118909 | 0,2567254 | 0,23770865 | 0,24953439 | 0,61360876 | 0,68850135 | 0,50927789 | 0,55171658 | 0,61746658 | 0,71220793 |
| -9,5 | 0,03584954 | 0,07992915 | 0,00960096 | 0,01158901 | 0,02281171 | 0,00622088 | 0,01716094 | 0,02817068 | 0,05182968 | 0,11376225 | 0,15307623 | 0,07771429 | 0,14856087 | 0,16581849 | 0,1407154 |
| -10 | 0,00171594 | 0,00850811 | 0 | 0 | 0 | 0 | 0 | 0,00136647 | 0 | 0,01181558 | 0,00576026 | 0,00563474 | 0,00342403 | 0,01118895 | 0,00619948 |

| centration log | | | | | | FPR2 | | | | | | | | | |
|---|---|---|---|---|---|---|---|---|---|---|---|---|---|---|---|
| -5,5 | 0,28172967 | 0,25534934 | 0,10296122 | 0,2331093 | 0,28083629 | 0,20555576 | 0,13102046 | 0,25276237 | 0,26461459 | 0,48701002 | 0,39065716 | 0,36695447 | 0,40098651 | 0,43992695 | 0,42681319 |
| -6 | 0,06714817 | 0,0709773 | 0,02155942 | 0,10501315 | 0,04500042 | 0,01333642 | 0,06711392 | 0,05832647 | 0,05834319 | 0,31163594 | 0,16415374 | 0,11986511 | 0,32872 | 0,22521982 | 0,28223569 |
| -6,5 | 0,05358563 | 0,03329152 | 0,0164788 | 0,00243036 | 0,00650729 | 0 | 0,0045191 | 0,00571472 | 0,01580668 | 0,08858035 | 0,0490805 | 0,0273257 | 0,04701718 | 0,07695017 | 0,06669953 |
| -7 | 0,00160926 | 0 | 0 | 0 | 0 | 0 | 0 | 0,00280315 | 0 | 0,01312151 | 0,00194764 | 0 | 0,02268438 | 0,00915314 | 0,00521301 |
| -7,5 | 0 | 0 | 0 | 0 | 0 | 0 | 0,0009285 | 0 | 0 | 0,00179876 | 0 | 0 | 0,00131193 | 0 | 0 |
| -8 | 0 | 0 | 0 | 0,00088014 | 0 | 0,00378297 | 0,00241426 | 0 | 0 | 0 | 0,00159874 | 0 | 0 | 0,00160123 | 0,00440379 |
| -8,5 | 0 | 0,00171766 | 0 | 0 | 0 | 0,00547051 | 0 | 0 | 0,00149945 | 0,00143777 | 0 | 0,00140705 | 0,00231298 | 0 | 0,00331637 |
| -9 | 0 | 0 | 0 | 0 | 0 | 0 | 0 | 0 | 0 | 0 | 0 | 0,0028946 | 0 | 0 | 0,00345236 |
| -9,5 | 0 | 0 | 0 | 0 | 0,00757652 | 0,00403091 | 0 | 0,01245115 | 0,00431091 | 0,00112606 | 0,00128245 | 0,00192438 | 0 | 0,00262647 | 0 |
| -10 | 0,00316188 | 0 | 0,0040833 | 0 | 0,001323 | 0 | 0,00103256 | 0,00269657 | 0 | 0 | 0 | 0 | 0 | 0 | 0 |

| centration log | | | | | | FPR3 | | | | | | | | | |
|---|---|---|---|---|---|---|---|---|---|---|---|---|---|---|---|
| -5,5 | 0,00210647 | 0 | 0,00639502 | 0,00502894 | 0,00557121 | 0,00104308 | 0 | 0,00086336 | 0,00179359 | 0,00229778 | 0,01034103 | 0,00157556 | 0 | 0,0055395 | 0,02169422 |
| -6 | 0 | 0 | 0,00093752 | 0 | 0,00097452 | 0,00119085 | 0 | 0,00103632 | 0,00110439 | 0,0035692 | 0 | 0 | 0,0035692 | 0,00110439 | 0 |
| -6,5 | 0 | 0 | 0 | 0 | 0 | 0,00086336 | 0 | 0,0014772 | 0,00109132 | 0,00567901 | 0 | 0,00106183 | 0,00109132 | 0,00208107 | 0,00106183 |
| -7 | 0 | 0 | 0 | 0 | 0 | 0,00103632 | 0 | 0 | 0 | 0 | 0,00165562 | 0,00172846 | 0,00373403 | 0 | 0,00172846 |
| -7,5 | 0 | 0,00212758 | 0 | 0 | 0 | 0,0014772 | 0,00093752 | 0,00179359 | 0,00157556 | 0,00179359 | 0 | 0 | 0 | 0,00157556 | 0 |
| -8 | 0 | 0 | 0,00393797 | 0 | 0,00093494 | 0,0020626 | 0 | 0 | 0,0012689 | 0 | 0 | 0,00406117 | | 0,0012689 | 0,00406117 |
| -8,5 | 0 | 0 | 0 | 0,00123892 | 0,00885044 | 0,00256068 | 0 | 0 | 0 | 0 | 0,00152394 | 0 | 0 | 0 | 0 |
| -9 | 0 | 0 | 0,00275153 | 0 | 0 | 0,00962544 | 0 | 0 | 0 | 0 | 0 | 0 | 0,001195 | 0 | 0 |
| -9,5 | 0 | 0,00174507 | 0,00095354 | 0,00210095 | 0,00441664 | 0,00208277 | 0,00174507 | 0 | 0,00559447 | 0 | 0 | 0,00128571 | 0 | 0,00559447 | 0,00128571 |
| -10 | 0 | 0 | 0,00160533 | 0 | 0,00151753 | 0,00507861 | 0 | 0 | 0 | 0 | 0 | 0 | 0 | 0 | 0 |

Supporting Fig 1c

Binding of f-MVPIK(TAMRA)I to FPR in HEK 293T

| concentration Log (M) | | | | | | | | | | | | | Mock | | | | | | | | | | | | |
|---|---|---|---|---|---|---|---|---|---|---|---|---|---|---|---|---|---|---|---|---|---|---|---|---|---|
| -5,5 | 0,04598834 | 0,13097613 | 0,24737583 | 0,02266987 | 0,02749564 | 0,03122433 | 0,01510349 | 0,00728233 | 0,01409285 | 0,02902232 | 0,010088 | 0,01243051 | 0,00054688 | 0,00598689 | 0,02479256 | 0,016081 | 0,01658323 | 0,02941654 | 0,00735655 | 0,00509568 | 0,00978642 | 0,01091932 | 0,01616015 | 0,00386915 | 0,00432915 |
| -6 | 0,11963973 | 0,0218175 | 0,01135638 | 0,01423978 | 0,01027552 | 0,00927939 | 0,00161706 | 0,0038147 | 0,01318284 | 0,01129289 | 0,00855295 | 0,00608559 | 0,0036222 | 0,00289952 | 0,01032882 | 0,00665756 | 0,0016985 | 0,00124414 | 0,00234448 | 0,00165182 | 0,01825972 | 0,01286715 | 0,00255266 | 0,00041106 | 0,00236564 |
| -6,5 | 0,00707573 | 0,00273923 | 0,00515065 | 0,01447468 | 0,00067803 | 0,00377497 | 0,00107274 | 0,00058988 | 0,00303647 | 0,00316129 | 0 | 0,00084847 | 0,00557654 | 0,00061873 | 0,00336348 | 0 | 0,00411516 | 0 | 0 | 0,00193769 | 0,00276788 | 0,00112484 | 0,00099079 | 0,00140337 | 0,00090706 |
| -7 | 0,00932861 | 0,0006913 | 0,00795795 | 0,01033014 | 0,00455559 | 0,00942097 | 0 | 0,0023366 | 0,00393824 | 0,00582974 | 0,00245372 | 0,00096654 | 0,00606298 | 0,00311835 | 0,00108056 | 0,00652624 | 0 | 0,00243685 | 0,00190631 | 0,00047162 | 0 | 0,00352057 | 0,00234178 | 0,0121184 | 0,00083365 |
| -7,5 | 0,02272158 | 0,00407518 | 0,00517546 | 0,00071919 | 0,00185947 | 0,00745745 | 0,00258279 | 0,00059506 | 0 | 0,01663193 | 0,00061289 | 0,00077211 | 0 | 0,00063216 | 0,00223235 | 0,01150525 | 0,00536689 | 0 | 0,00088817 | 0,0010065 | 0 | 0,00095573 | 0,00859838 | 0,00290795 | 0,00341652 |
| -8 | 0 | 0,00342275 | 0,00551355 | 0,00243695 | 0,00121004 | 0,00610177 | 0,00812065 | 0,00063857 | 0,0033533 | 0 | 0,0005952 | 0,00767638 | 0,00987294 | 0,00138281 | 0,03811118 | 0 | 0,00548538 | 0,00568597 | 0,00216662 | 0,00839519 | 0,00163653 | 0,00638025 | 0,00613717 | 0,00043868 | 0 |
| -8,5 | 0,00213596 | 0,00597263 | 0,0005579 | 0,02954047 | 0,00387763 | 0,00554946 | 0,00630519 | 0 | 0,0006462 | 0,00598607 | 0,00254992 | 0,00096718 | 0,00827079 | 0 | 0,0024817 | 0,00093469 | 0,00423889 | 0,00195815 | 0,00260471 | 0,00044535 | 0,00323339 | 0,00529137 | 0 | 0,00587214 | 0,00264459 |
| -9 | 0,00289998 | 0,00996915 | 0,00478047 | 0,01128638 | 0,00211355 | 0,00772191 | 0 | 0,00476399 | 0,01200063 | 0,00966854 | 0,00366593 | 0,00250281 | 0,00170791 | 0,00377904 | 0 | 0,00097346 | 0,00113514 | 0,00339833 | 0,00231097 | 0,00092688 | 0,00399744 | 0,00103818 | 0 | 0 | 0 |
| -9,5 | 0,00417552 | 0 | 0,00420494 | 0,00489769 | 0,00731866 | 0,00935951 | 0,00348953 | 0 | 0,00116585 | 0,0113561 | 0,00555043 | 0,00200897 | 0,00100078 | 0 | 0,0005321 | 0,03796678 | 0,01300041 | 0,00355062 | 0,00522018 | 0,00523482 | 0,00535815 | 0,63086544 | 0,00235427 | 0,00092473 | 0 |
| -10 | | | | 0,00344085 | 0,00382581 | 0,0081247 | 0,00513288 | 0 | 0,0044991 | 0,00955229 | 0,00148928 | 0,00070528 | 0,01195075 | 0 | 0,00285739 | 0 | 0,00684831 | 0 | 0 | 0,0018339 | 0,00084217 | 0,00248255 | 0,0014978 | 0,00044068 | 0,00121084 |

| concentration Log (M) | | | | | | | | | | | | | FPR1 | | | | | | | | | | | | |
|---|---|---|---|---|---|---|---|---|---|---|---|---|---|---|---|---|---|---|---|---|---|---|---|---|---|
| | 0,94599014 | 0,89023767 | 0,87662637 | 0,92033649 | 0,73175167 | 0,87024283 | 0,59283511 | 0,80665769 | 0,74949642 | 0,83736879 | 0,71243275 | 0,84385034 | 0,66856379 | 0,82694716 | 0,57415726 | 0,91879037 | 0,98580156 | 0,8648541 | 1 | 1 | 0,99373492 | 1 | 0,92053754 | 1 | 0,93235963 |
| | 0,99213983 | 0,89028201 | 0,81708988 | 0,86664121 | 0,79232122 | 0,90066875 | 0,97185423 | 1 | 0,79787372 | 0,96043091 | 0,74799136 | 0,72845066 | 0,88494708 | 0,98762742 | 0,86925564 | 1 | 1 | 1 | 0,87394687 | 0,94453208 | 1 | 0,93361037 | 1 | 0,86320729 | 0,85826364 |
| | 1 | 0,93453549 | 0,91788859 | 0,82187582 | 0,93578293 | 0,78237051 | 0,85324579 | 0,89820565 | 0,88866654 | 1 | 0,88928724 | 0,8820658 | 1 | 0,9499277 | 0,89167406 | 0,85273048 | 0,78121727 | 0,66230747 | 0,93526178 | 0,9284654 | 0,85981956 | 0,80707369 | 0,75755811 | 0,96521694 | 1 |
| | 0,93880754 | 1 | 0,84879628 | 0,95543685 | 0,89835691 | 0,93257174 | 0,97991525 | 0,99124119 | 1 | 0,97422248 | 0,8645484 | 0,91576317 | 0,87500606 | 0,99103461 | 1 | 0,86290476 | 0,95195835 | 0,771843 | 0,84714719 | 0,77382596 | 0,83913641 | 0,98382257 | 0,92197701 | 0,90666446 | 0,9289248 |
| | 0,97340256 | 0,87077048 | 1 | 0,96650707 | 1 | 0,99308268 | 1 | 0,99788222 | 0,90610146 | 0,9826715 | 1 | 1 | 0,9398677 | 1 | 0,940349 | 0,88040885 | 0,85822332 | 0,79046027 | 0,87425044 | 0,84518184 | 0,84875322 | 0,89951973 | 0,90186395 | 0,85990012 | 0,72180766 |
| | 0,74977425 | 0,63285647 | 0,52804297 | 1 | 0,92532619 | 1 | 0,79664408 | 0,93536279 | 0,85363338 | 0,92438617 | 0,88589519 | 0,90407822 | 0,80868134 | 0,97589297 | 0,84793049 | 0,7418439 | 0,7131201 | 0,52198804 | 0,52689756 | 0,59935384 | 0,42313413 | 0,72927957 | 0,72883378 | 0,61413095 | 0,57845726 |
| | 0,79110819 | 0,62068042 | 0,59504126 | 0,6557433 | 0,56176743 | 0,5977976 | 0,65438317 | 0,66565119 | 0,63467654 | 0,67192546 | 0,53219632 | 0,67114223 | 0,58826232 | 0,70110362 | 0,69550343 | 0,52566903 | 0,5253081 | 0,41922007 | 0,47989403 | 0,46126975 | 0,47960527 | 0,59793092 | 0,586947 | 0,48321769 | 0,52690512 |
| | 0,61719307 | 0,4739755 | 0,38165952 | 0,36268196 | 0,34248559 | 0,3983604 | 0,39232633 | 0,44066549 | 0,53212546 | 0,3844681 | 0,3345626 | 0,32461571 | 0,42364901 | 0,45241344 | 0,42097969 | 0,46123488 | 0,43940565 | 0,43475072 | 0,39057909 | 0,46407943 | 0,3915907 | 0,47986102 | 0,57568778 | 0,34984332 | 0,34896796 |
| | 0,5521842 | 0,50865163 | 0,42310186 | 0,09124412 | 0,03496572 | 0,04153137 | 0,06231046 | 0,05991849 | 0,04476496 | 0,07469078 | 0,03355669 | 0,03445694 | 0,07113402 | 0,08082295 | 0,0445526 | 0,25732673 | 0,19882937 | 0,19693275 | 0,28961611 | 0,27806606 | 0,29013829 | 0,20687066 | 0,27042886 | 0,37448555 | 0,39128596 |
| | | | | 0,00357064 | 0,0027045 | 0 | 0,0166854 | 0,00522353 | 0,00148381 | 0,00617179 | 0,0010546 | 0,00372386 | 0,00781308 | 0,00431558 | 0,00521346 | 0,04226277 | 0,0377132 | 0,03341662 | 0,0420473 | 0,07635268 | 0,05679403 | 0,07764177 | 0,03787845 | 0,0454435 | 0,05656332 |

| concentration Log (M) | | | | | | | | | | | | | FPR2 | | | | | | | | | | | | |
|---|---|---|---|---|---|---|---|---|---|---|---|---|---|---|---|---|---|---|---|---|---|---|---|---|---|
| | 0,76248611 | 0,63311351 | 0,66840345 | 0,3948436 | 0,33532565 | 0,45040267 | 0,3036718 | 0,43112865 | 0,35046912 | 0,39918251 | 0,25149508 | 0,23281683 | 0,24411754 | 0,26525227 | 0,16139025 | 0,88495568 | 0,73736044 | 0,64257528 | 0,55466357 | 0,57776751 | 0,67727874 | 0,68602887 | 0,817396 | 0,68945254 | 0,63395846 |
| | 0,60483006 | 0,65324496 | 0,56619366 | 0,47246373 | 0,40257984 | 0,41842238 | 0,35059752 | 0,29547368 | 0,35454628 | 0,48299095 | 0,32061433 | 0,17244097 | 0,21792348 | 0,21871584 | 0,13521252 | 0,60986993 | 0,66492949 | 0,54874076 | 0,74194574 | 0,7469629 | 0,66302782 | 0,69245607 | 0,68648648 | 0,81895632 | 0,54021163 |
| | 0,43170182 | 0,35896306 | 0,25601548 | 0,11853108 | 0,05867792 | 0,06078289 | 0,13450319 | 0,10987656 | 0,13794624 | 0,08591157 | 0,04873872 | 0,07475356 | 0,12676315 | 0,06305932 | 0,10315285 | 0,30259698 | 0,32984869 | 0,27845358 | 0,35363846 | 0,30659667 | 0,3447098 | 0,24305972 | 0,26945325 | 0,32383052 | 0,31324467 |
| | 0,17028121 | 0,10450084 | 0,11332658 | 0,02562591 | 0,02170958 | 0,03480411 | 0,02615107 | 0,01847444 | 0,03543043 | 0,0381322 | 0,01690146 | 0,0170105 | 0,02553249 | 0,01798482 | 0,01045828 | 0,11216952 | 0,07465418 | 0,07606109 | 0,04325473 | 0,04900323 | 0,03854136 | 0,10701212 | 0,12763834 | 0,08314717 | 0,09319864 |
| | 0,01605456 | 0,01870402 | 0,00375977 | 0,0072338 | 0,0026132 | 0,00401971 | 0 | 0,00247169 | 0,00378857 | 0,00515025 | 0,00204901 | 0 | 0,01576365 | 0,00193562 | 0,00157364 | 0,00179631 | 0,00216382 | 0,001696 | 0,00231 | 0,00384411 | 0,00397532 | 0,00899747 | 0,00297289 | 0,01410238 | 0,00227768 |
| | 0,00523264 | 0,01021291 | 0,00235847 | 0,01434823 | 0,0039297 | 0,00448492 | 0,00380323 | 0 | 0,01674893 | 0 | 0,00168065 | 0 | 0,00477614 | 0,00275124 | 0,00199328 | 0,02351117 | 0,00047801 | 0,00081007 | 0,00119086 | 0,0022714 | 0,00206886 | 0 | 0,00212871 | 0,00174279 | 0,00228374 |
| | 0,01327434 | 0,00063189 | 0,0017669 | 0,00703197 | 0,00192453 | 0,01387589 | 0,00559557 | 0,02570441 | 0,00111871 | 0,00113385 | 0,00470407 | 0,00470657 | 0,00057689 | 0,0088811 | 0,00107559 | 0,00796918 | 0,0088948 | 0,00304487 | 0,00155415 | 0 | 0,00169659 | 0 | 0,00876057 | 0,007697 | 0,00040716 |
| | 0,00382045 | 0,00314747 | 0 | 0,00857498 | 0,00051189 | 0,0027998 | 0,00313991 | 0,01396645 | 0,00064191 | 0,00315788 | 0 | 0,00962715 | 0,00461151 | 0,01144364 | 0,01472908 | 0,00511144 | 0 | 0,0004839 | 0 | 0,00229559 | 0 | 0,00118356 | 0 | 0 | 0 |
| | 0,00128288 | 0 | 0,01222093 | 0,00272708 | 0,00092736 | 0,00293637 | 0 | 0,00108627 | 0,00348954 | 0,00058484 | 0,00181909 | 0,0049191 | 0,00761949 | 0 | 0,00056233 | 0,00085665 | 0,00038737 | 0,00041471 | 0 | 0 | 0,00283085 | 0,00395082 | 0,0004338 | 0 | 0 |
| | | | | 0,00558179 | 0,00224193 | 0,00831019 | 0,00100906 | 0,00172944 | 0,00243121 | 0,00888476 | 0,00223905 | 0,00170188 | 0,00545257 | 0,00125097 | 0,00574554 | 0,00047357 | 0,0015558 | 0,00301694 | 0,00046103 | 0,00247755 | 0,00052226 | 0 | 0,07347159 | 0,00113715 | 0,0026264 |

| concentration Log (M) | | | | | | | | | | | | | FPR3 | | | | | | | | | | | | |
|---|---|---|---|---|---|---|---|---|---|---|---|---|---|---|---|---|---|---|---|---|---|---|---|---|---|
| | 0,39455161 | 0,41563793 | 0,3994556 | 0,12856776 | 0,16177759 | 0,1556042 | 0,07109271 | 0,20547724 | 0,08753401 | 0,12916934 | 0,13745188 | 0,05557724 | 0,13348143 | 0,14507259 | 0,07829323 | 0,04026084 | 0,03659775 | 0,08443342 | 0,11062235 | 0,08325819 | 0,05486499 | 0,08975913 | 0,07427652 | 0,07811316 | 0,05366359 |
| | 0,17594752 | 0,14048601 | 0,11614414 | 0,06300745 | 0,08717195 | 0,04969937 | 0,02994821 | 0,01788461 | 0,0595958 | 0,02533458 | 0,06279991 | 0,0478454 | 0,04173248 | 0,01470729 | 0,05040399 | 0,00894017 | 0,01746454 | 0,02769258 | 0,05314264 | 0,05418959 | 0,05478941 | 0,03490357 | 0,0116291 | 0,02222901 | 0,02633568 |
| | 0,01866875 | 0,03790315 | 0,02802237 | 0,01368577 | 0,0191544 | 0,00539802 | 0,0142472 | 0,01358077 | 0,00691438 | 0,00679074 | 0,00536626 | 0,00770884 | 0,0012043 | 0,0110536 | 0,01035397 | 0,00129351 | 0,01360551 | 0,00153139 | 0,02527077 | 0,02945346 | 0,01379324 | 0,00570102 | 0,00265228 | 0,00754791 | 0,003688 |
| | 0,00190956 | 0,00525735 | 0,02790488 | 0,00100847 | 0,01246132 | 0 | 0,01214014 | 0,00191797 | 0,00284275 | 0,00478853 | 0,00585808 | 0,004966 | 0,00858939 | 0,00068181 | 0,00156025 | 0,00131458 | 0,00238238 | 0 | 0,0004699 | 0,01178563 | 0,00420884 | 0 | 0,00102725 | 0,00236141 | 0,0042872 |
| | 0,00549861 | 0,02463482 | 0,00192134 | 0,0101715 | 0,00050502 | 0,00168315 | 0 | 0,10412022 | 0,00057417 | 0,00059459 | 0,0004975 | 0,00159284 | 0,00564812 | 0,00323454 | 0,0054118 | 0,00480677 | 0,00353182 | 0,00052224 | 0,0018909 | 0 | 0,00588672 | 0,0071302 | 0,01152964 | 0,00083822 | 0,01077369 |
| | 0,00337812 | 0,00502554 | 0,00388496 | 0,00230576 | 0 | 0,00105128 | 0,00260292 | 0,00219332 | 0,00260565 | 0,00131248 | 0 | 0,00828364 | 0,0012372 | 0,00310399 | 0,01376998 | 0 | 0,00348866 | 0,00170321 | 0 | 0,0023688 | 0,00296633 | 0,00072202 | 0,00122689 | 0 | 0,00266384 |
| | 0,00525146 | 0,00062518 | 0,00784076 | 0,01094089 | 0,00808434 | 0,00770745 | 0,04869943 | 0,00881864 | 0,00352015 | 0,00898289 | 0,00525315 | 0,00167005 | 0,00055429 | 0,0126336 | 0,00411928 | 0 | 0 | 0,00318563 | 0,00042784 | 0,00089727 | 0 | 0 | 0,00250698 | 0,00411146 | 0,00057422 |
| | 0,00925103 | 0,0066074 | 0,00740006 | 0,00051334 | 0,00143448 | 0,00048923 | 0,00913213 | 0,00466146 | 0,00212375 | 0,00835783 | 0,001903 | 0,02145661 | 0,00218508 | 0,00930777 | 0,00049204 | 0,00058049 | 0,00159269 | 0,0014736 | 0 | 0,00230041 | 0 | 0 | 0 | 0,00384023 | 0,00228705 |
| | 0,00189568 | 0,0012231 | 0,00334859 | 0,00412869 | 0,00578758 | 0,0009735 | 0,0107559 | 0,00961254 | 0 | 0,00336889 | 0 | 0,00148676 | 0,00177879 | 0,00600571 | 0,00253658 | 0,00289051 | 0,00047701 | 0,00231068 | 0,00346864 | 0,00474787 | 0 | 0 | 0,00135263 | 0,00102067 | 0,00071573 |
| | | | | 0,0042654 | 0,00354545 | 0,00149407 | 0,00316512 | 0,00407585 | 0,00118923 | 0,00268867 | 0,00303749 | 0,0166543 | 0,00108689 | 0,00612557 | 0,00114389 | 0,00161012 | 0,00101824 | 0,00159308 | 0 | 0,00189709 | 0,0039838 | 0,00270548 | 0 | 0,00133708 | 0,00242522 |

**Supporting Fig 2a :**

**Total fluorescence normalized to total cell number**

| 30min | 30min | 30min | 15min | 15min | 15min | 5min | 5min | 5min | 1min | 1min | 1min | | |
|---|---|---|---|---|---|---|---|---|---|---|---|---|---|
| 9556,42396 | 9532,47153 | 11310,6611 | 11816,7672 | 4984,27754 | 14008,7303 | 8862,80885 | 10320,1817 | 11262,1038 | 14619,8504 | 8212,06088 | 9473,93446 | Mock | |
| 2227193,63 | 433155,92 | 791811,699 | 3654685,94 | 2208823,87 | 2464471,09 | 3815900,14 | 8435641,54 | 3661880,79 | 148516,652 | 446199,561 | 224779,794 | FPR1 HEK test | fMVPI(FITC) |
| 9088256,33 | 5652712,3 | 3390545,14 | 1981214,57 | 7443231,37 | 6959225,65 | 2221354,46 | 6538505,07 | 4985485,96 | 340984,047 | 306193,259 | 272305,768 | FPR1 HEK test | fMVPI(FITC) |
| 2647105,02 | 5467962,09 | 2783322,87 | 2267017,52 | 2095484,85 | 5999985,16 | 6090277,63 | 6099588,61 | 2012409,95 | 115109,982 | 170364,97 | 547934,234 | FPR1 HEK test | fMVPI(FITC) |
| 10165754,4 | 7936888,49 | 8208525,58 | 7968714,32 | 6366716,25 | 6668930,13 | 7508955,26 | 5932633,15 | 5929556,19 | 5808962,84 | 7093471,16 | 7847811,58 | FPR1 HEK test | fMVPI(FITC) |
| 9848118,28 | 1884593,93 | 3651168,94 | 2441514,68 | 6438370,87 | 4154984,6 | 7666527,37 | 1136813,34 | 712428,069 | 4280282,48 | 5887291,65 | 7394275,69 | FPR1 HEK test | fMVPI(FITC) |
| 9099040,66 | 4668911,54 | 4625892,68 | 6724290,79 | 2598344,35 | 4504401,87 | 6667685,65 | 8074200,61 | 6177261,88 | 4694460,15 | 3561670,73 | 6061764,83 | FPR1 HEK test | fMVPI(FITC) |
| 575940,512 | 6492685,43 | 3290653,57 | 4592085,11 | 4009929,64 | 7080192,72 | 7612904,9 | 8624404,54 | 7428834,87 | 1443912,79 | 9720,38076 | 2295318,36 | FPR1 HEK test | fMVPI(FITC) |

I 1µM
I 1µM
I 1µM
I 1µM
I 1µM
I 1µM
I 1µM

Supporting Fig 2b:

Total fluorescence normalized to total cell number

| | | | | | | | | | | | | |
|---|---|---|---|---|---|---|---|---|---|---|---|---|
| n=1 | Mock | Mock | Mock | 30 | 30 | 30min | 4h | 4h | 4h | 24h | 24h | 24h |
| | 4460,88962 | 5454,71173 | 5506,90937 | 221455,871 | 1475766,4 | 2531843,59 | 1426924,07 | 196097,076 | 1162510,91 | 564943,941 | 105320,271 | 1349600,2 |
| | 3345,30732 | 3313,1708 | 4683,59998 | 3235314 | 217742,208 | 209431,992 | 454318,479 | 1356271,04 | 477175,481 | 246984,814 | 1275416,76 | 302158,384 |
| | 6435,19224 | 9139,35137 | 11127,4212 | 2036514,25 | 112193,824 | 532209,323 | 1295389,7 | 141837,083 | 143658,436 | 1343219,98 | 72914,8995 | 62915,7984 |
| | | | | | | | | | | | | |
| | Mock | Mock | Mock | 30 | 30 | 30min | 4h | 4h | 4h | 24h | 24h | 24h |
| n=2 | 8654,14212 | 9491,11824 | | 7617,30681 | 429387,569 | 872238,117 | 1636780,58 | 6467,85905 | 294822,11 | 88008,1541 | 5002,32226 | 36740,3568 |
| | 7100,9634 | 9113,37405 | 5539,00842 | 3536,55301 | 1504926,79 | 702807,698 | 161483,745 | 327100,173 | 2014299,34 | 47628,1903 | 146076,324 | 563011,952 |
| | | | | 6201,98457 | 132104,98 | 755579,443 | 238045,36 | 769603,447 | 67139,9761 | 111539,27 | 405114,737 | 140994,618 |
| | | | | 29688,5169 | 104061,577 | 126596,65 | 221315,921 | 115381,314 | 939813,823 | 1127726,44 | 240120,293 | 1398430,66 |
| | | | | 372896,655 | 511124,475 | 474622,719 | 99414,548 | 324532,267 | 501946,118 | 153703,896 | 219722,802 | |
| | | | | | | | 163214,775 | 524347,637 | | 158194,577 | 149125,195 | |
| | | | | | | | | | | | | |
| | Mock | Mock | Mock | 30 | 30 | 30min | 4h | 4h | 4h | 24h | 24h | 24h |
| n=3 | 6827,5906 | 12434,9102 | 10501,6838 | 48776,0777 | 4924,57909 | 4831,85808 | 19085,706 | 6342,89045 | 16101,6813 | 18885,5111 | 10926,1431 | 7834,69538 |
| | 12136,8671 | | 19957,0802 | 13883,6113 | 11553,6842 | 12074,2951 | 19753,7249 | 17019,8229 | 13729,3217 | 22145,7684 | 19996,6784 | 18325,3363 |
| | | | | 22864,4437 | 21552,0077 | 13360,2549 | 17111,4606 | 25466,0775 | 5489,50192 | 18994,2046 | 34617,4809 | |
| | | | | 15733,5325 | 30035,9201 | 16795,1978 | 15041,1403 | 27714,7985 | 10812,9171 | 23946,1621 | 28684,1519 | |

Supporting Fig 2c

Endocytosis f-MVPIK(FITC)I in HEK293T cells

concentration -log(M) Mock

| | | | | | | | | | | |
|---|---|---|---|---|---|---|---|---|---|---|
| -5,5 | 0,00844595 | 0,02711864 | 0,02654867 | 0,02828619 | 0,02998236 | 0 | 0,01463415 | 0,01675978 | 0,01169591 | 0,02863436 |
| -6 | 0,02313167 | 0,01497504 | 0,01883562 | 0,01092896 | 0,01715266 | 0 | 0,02994012 | 0,03076923 | 0,03389831 | 0,03183024 |
| -6,5 | 0,01624549 | 0,03136531 | 0,01217391 | 0,0141844 | 0,0177305 | 0 | 0 | 0,03164557 | 0,00442478 | 0,00831025 |
| -7 | 0 | 0,00357143 | 0,01196581 | 0 | 0 | 0,01666667 | 0,02013423 | 0,02109705 | 0,05288462 | 0,02369668 |
| -7,5 | 0,0019802 | 0,02641509 | 0,0224525 | 0,00352113 | 0,00580271 | 0,01785714 | 0,01538462 | 0,01546392 | 0,00688073 | 0,00806452 |
| -8 | 0,01782531 | 0,02364865 | 0,01158301 | 0,01811594 | 0 | 0 | 0 | 0 | 0,00680272 | 0,0131579 |
| -8,5 | 0,02044293 | 0,01151632 | 0,01567944 | 0,02014652 | 0,01906413 | 0 | 0 | 0,03333333 | 0,01030928 | 0,00288184 |
| -9 | 0,01196581 | 0,02763385 | 0,02654867 | 0,01554404 | 0,00801603 | 0 | 0 | 0 | 0,01114206 | 0,00282486 |

FPR1

concentration -log(M)

| | | | | | | | | | | |
|---|---|---|---|---|---|---|---|---|---|---|
| -5,5 | 2,12562814 | 2,38947368 | 2,25085911 | 2,2733564 | 3,20599251 | 1,91139241 | 3,19879518 | 3,48701299 | 1,71875 | 3,37309645 |
| -6 | 2,40884956 | 1,98453608 | 2,44596913 | 1,65483235 | 3,00747664 | 2,15238095 | 2,46842105 | 2,23931624 | 2,44759207 | 3,43062201 |
| -6,5 | 1,24767225 | 2,23172906 | 2,12749004 | 1,25776965 | 2,15267176 | 1,89534884 | 1,46 | 2,59313726 | 2,50645995 | 2,33707865 |
| -7 | 1,11133201 | 1,58287796 | 2,11208791 | 2,21139706 | 2,91666667 | 1,47204969 | 1,75333333 | 2,05652174 | 2,2235023 | 3,4005305 |
| -7,5 | 1,86948854 | 2,42474916 | 2,21287129 | 2,31150443 | 2,46701389 | 1,83695652 | 1,69607843 | 2,04455446 | 1,60635697 | 1,99036145 |
| -8 | 2,28979592 | 3,02729045 | 2,51888668 | 2,10204082 | 2,12189055 | 1,97478992 | 0,7752809 | 0,50847458 | 1,46190476 | 1,45647059 |
| -8,5 | 1,36286201 | 1,64554795 | 1,375 | 1,503663 | 1,3890785 | 0,3 | 0,42105263 | 0,37931035 | 0,56701031 | 0,64012739 |
| -9 | 0,81487603 | 1,09722222 | 0,93065693 | 0,64173913 | 0,16377171 | 0,08695652 | 0 | 0 | 0,3454039 | 0,11351351 |

concentration -log(M) FPR2

| | | | | | | | | | | |
|---|---|---|---|---|---|---|---|---|---|---|
| -5,5 | 1,24064171 | 1,19821429 | 1,33222037 | 0,98070175 | 1,46192893 | 1,38888889 | 1,375 | 1,62676056 | 1,04522613 | 1,33701658 |
| -6 | 0,47069272 | 0,67358491 | 0,45315488 | 0,34707904 | 0,6331361 | 0,58119658 | 0,86486487 | 0,63809524 | 0,5984252 | 0,59493671 |
| -6,5 | 0,04844291 | 0,14206642 | 0,08704062 | 0,09532374 | 0,15700483 | 0,26174497 | 0,2278481 | 0,22543353 | 0,11311054 | 0,18703242 |
| -7 | 0,07913669 | 0,0539629 | 0,08167331 | 0,05753968 | 0,05762082 | 0,06837607 | 0,12041885 | 0,12041885 | 0,04418605 | 0,03309693 |
| -7,5 | 0,0231405 | 0,05016722 | 0,05369128 | 0,02 | 0,03384095 | 0,05660377 | 0,04651163 | 0,05714286 | 0,02666667 | 0,02624672 |
| -8 | 0,03818182 | 0,05389222 | 0,04291846 | 0,0627451 | 0,07716049 | 0,02013423 | 0,03092784 | 0 | 0,00932401 | 0,02191781 |
| -8,5 | 0,02504174 | 0,04137931 | 0,03853955 | 0,06398538 | 0,02777778 | 0,00934579 | 0,01724138 | 0,0173913 | 0,0173913 | 0,01785714 |
| -9 | 0,01785714 | 0,01565217 | 0,02905983 | 0,03956044 | 0 | 0 | 0 | 0 | 0,00277778 | 0,00536193 |

Support Fig 3b and 4b calicum signal in HEK293T cells

HEK cells Calicum signal (dF/F0)

| Log Concentration (M) | | | Mock | | | FPR1 | | | | | | FPR2 | | | | FPR3 | |
|---|---|---|---|---|---|---|---|---|---|---|---|---|---|---|---|---|---|
| -6 | 0,1 | 0,2 | 0,17 | 0,93 | 0,8 | 0,75 | 0,94 | 1,4 | 0,88 | 0,63 | 0,7 | 0,64 | 0,57 | 0,29 | 0,29 | 0,14 | 0,38 |
| -6,5 | 0,16 | 0,3 | 0,11 | 0,67 | 0,76 | 0,85 | 0,92 | 0,92 | 0,64 | 0,54 | 0,68 | 0,66 | 0,55 | 0,21 | 0,21 | 0,24 | 0,23 |
| -7 | 0,15 | 0,1 | 0,18 | 0,61 | 0,67 | 0,57 | 0,86 | 0,86 | 0,57 | 0,45 | 0,48 | 0,45 | 0,4 | 0,29 | 0,29 | 0,23 | 0,28 |
| -7,5 | 0,16 | 0,1 | 0,14 | 0,46 | 0,66 | 0,71 | 0,76 | 0,67 | 0,44 | 0,26 | 0,3 | 0,3 | 0,23 | 0,19 | 0,19 | 0,16 | 0,17 |
| -8 | 0,14 | 0,1 | 0,11 | 0,44 | 0,5 | 0,65 | 0,63 | 0,53 | 0,36 | 0,2 | 0,19 | 0,27 | 0,18 | 0,17 | 0,17 | 0,14 | 0,16 |
| -8,5 | 0,14 | 0,17 | 0,15 | 0,45 | 0,49 | 0,27 | 0,32 | 0,31 | 0,35 | 0,13 | 0,15 | 0,21 | 0,13 | 0,15 | 0,15 | 0,17 | 0,14 |
| -9 | 0,11 | 0,1 | 0,08 | 0,23 | 0,31 | 0,18 | 0,24 | 0,24 | 0,29 | 0,12 | 0,17 | 0,18 | 0,09 | 0,13 | 0,13 | 0,09 | 0,07 |
| -9,5 | 0,02 | 0,06 | 0,09 | 0,07 | 0,06 | 0,09 | 0,08 | 0,09 | 0,06 | 0,01 | 0,01 | 0,01 | 0,07 | 0,09 | 0,02 | 0,02 | 0,09 |

FPR1 -HEK Calicum signal (dF/F0)

| Log Concentration (M) | | | f-MVPIKI | | | f-MVPIK(Ahx-DOTA)I | | | f-MVPIK(Ahx-DOTA-Lu)I | | | f-MVPIK(Ahx-DOTA-Ga)I |
|---|---|---|---|---|---|---|---|---|---|---|---|---|
| -6 | 0,75 | 0,94 | 0,85 | 0,6 | 0,87 | 0,706 | 0,93 | 0,87 | 0,9 | 0,85 | 0,89 | 0,8 |
| -6,5 | 0,85 | 0,92 | 0,85 | 0,7 | 0,64 | 0,7 | 0,91 | 0,74 | 0,82 | 0,85 | 0,8 | 0,68 |
| -7 | 0,57 | 0,86 | 0,73 | 0,68 | 0,58 | 0,65 | 0,89 | 0,6 | 0,71 | 0,74 | 0,87 | 0,65 |
| -7,5 | 0,71 | 0,77 | 0,68 | 0,54 | 0,43 | 0,45 | 0,7 | 0,5 | 0,6 | 0,66 | 0,52 | 0,6 |
| -8 | 0,65 | 0,63 | 0,6 | 0,29 | 0,31 | 0,27 | 0,58 | 0,41 | 0,43 | 0,28 | 0,27 | 0,3 |
| -8,5 | 0,27 | 0,32 | 0,34 | 0,25 | 0,28 | 0,23 | 0,31 | 0,28 | 0,34 | 0,12 | 0,27 | 0,23 |
| -9 | 0,18 | 0,24 | 0,22 | 0,12 | 0,23 | 0,14 | 0,13 | 0,23 | 0,2 | 0,14 | 0,13 | 0,12 |
| -9,5 | 0,07 | 0,06 | 0,09 | 0,08 | 0,09 | 0,06 | 0,06 | 0,09 | 0,08 | | | |

U87 cells Calicum signal (dF/F0)

| Log Concentration (M) | | | | f-MVPIKI | | | | f-MVPIK(Ahx-DOTA)I | | | f-MVPIK(Ahx-DOTA-Lu)I | | | | | | | f-MVPIK(Ahx-DOTA-Ga)I | | |
|---|---|---|---|---|---|---|---|---|---|---|---|---|---|---|---|---|---|---|---|---|
| -6 | 1,22 | 1,13 | 1,2 | 1,14 | 1,1 | 1,27 | 1,33 | 1,2 | 1,23 | 1,2 | 1,39 | 1,4 | 1,3 | 1,55 | 1,9 | 1,22 | 1,2 | 0,96 | 1,2 | 1,41 |
| -6,5 | 1,19 | 0,99 | 1,7 | 1,4 | 1,19 | 1,12 | 1,8 | 0,96 | 0,95 | 0,94 | 1,2 | 1,34 | 1 | 1,14 | 0,83 | 1,2 | 1,8 | 0,91 | 1,18 | 1,37 |
| -7 | 1 | 1,1 | 1,2 | 1,18 | 0,99 | 1,25 | 0,99 | 1,38 | 0,99 | 1,18 | 0,98 | 0,94 | 1,8 | 0,96 | 0,98 | 0,98 | 0,86 | 0,8 | 1,02 | 0,95 |
| -7,5 | 1 | 0,95 | 0,98 | 1,12 | 1,1 | 1,13 | 0,9 | 1,4 | 0,94 | 1 | 0,86 | 0,93 | 0,8 | 0,82 | 0,85 | 0,77 | 0,6 | 0,61 | 0,7 | 0,6 |
| -8 | 0,9 | 0,98 | 0,94 | 1,3 | 1,07 | 0,99 | 0,84 | 0,88 | 0,93 | 0,95 | 0,71 | 0,66 | 0,58 | 0,5 | 0,63 | 0,45 | 0,46 | 0,46 | 0,65 | 0,58 |
| -8,5 | 0,66 | 0,69 | 0,6 | 0,66 | 0,47 | 0,51 | 0,41 | 0,47 | 0,53 | 0,57 | 0,62 | 0,62 | 0,55 | 0,62 | 0,58 | 0,4 | 0,38 | 0,33 | 0,46 | 0,35 |
| -9 | 0,14 | 0,23 | 0,2 | 0,12 | 0,38 | 0,37 | 0,26 | 0,22 | 0,14 | 0,12 | 0,12 | 0,21 | 0,12 | 0,14 | 0,18 | 0,11 | 0,11 | 0,17 | 0,14 | 0,13 |
| -9,5 | 0,01 | 0,08 | 0,09 | 0,1 | 0,3 | 0,05 | 0,1 | 0,07 | 0,08 | 0,08 | 0,06 | 0,06 | 0,07 | 0,09 | 0,01 | 0,04 | 0,02 | 0,01 | 0,02 | 0,02 |

Supporting Fig 4 b: U87 cells Calicum  signal (dF/F0)

| Log Concentration (M | f-MVPIKI | | | | f-MVPIK(Ahx-DOTA)I | | | |
|---|---|---|---|---|---|---|---|---|
| -6 | 1,22 | 1,13 | 1,2 | 1,14 | 1,1 | 1,27 | 1,33 | 1,2 |
| -6,5 | 1,19 | 0,99 | 1,7 | 1,4 | 1,19 | 1,12 | 1,8 | 0,96 |
| -7 | 1 | 1,1 | 1,2 | 1,18 | 0,99 | 1,25 | 0,99 | 1,38 |
| -7,5 | 1 | 0,95 | 0,98 | 1,12 | 1,1 | 1,13 | 0,9 | 1,4 |
| -8 | 0,9 | 0,98 | 0,94 | 1,3 | 1,07 | 0,99 | 0,84 | 0,88 |
| -8,5 | 0,66 | 0,69 | 0,6 | 0,66 | 0,47 | 0,51 | 0,41 | 0,47 |
| -9 | 0,14 | 0,23 | 0,2 | 0,12 | 0,38 | 0,37 | 0,26 | 0,22 |
| -9,5 | 0,01 | 0,08 | 0,09 | 0,1 | 0,3 | 0,05 | 0,1 | 0,07 |

| Log Concentration (M | f-MVPIK(Ahx-DOTA-Lu)I | | | | | | | f-MVPIK(Ahx-DOTA-Ga)I | | | | |
|---|---|---|---|---|---|---|---|---|---|---|---|---|
| -6 | 1,23 | 1,2 | 1,39 | 1,4 | 1,3 | 1,55 | 1,9 | 0,96 | 1,2 | 1,41 | 1,22 | 1,2 |
| -6,5 | 0,95 | 0,94 | 1,2 | 1,34 | 1 | 1,14 | 0,83 | 0,91 | 1,18 | 1,37 | 1,2 | 1,8 |
| -7 | 0,99 | 1,18 | 0,98 | 0,94 | 1,8 | 0,96 | 0,98 | 0,8 | 1,02 | 0,95 | 0,98 | 0,86 |
| -7,5 | 0,94 | 1 | 0,86 | 0,93 | 0,8 | 0,82 | 0,85 | 0,61 | 0,7 | 0,6 | 0,77 | 0,6 |
| -8 | 0,93 | 0,95 | 0,71 | 0,66 | 0,58 | 0,5 | 0,63 | 0,46 | 0,65 | 0,58 | 0,45 | 0,46 |
| -8,5 | 0,53 | 0,57 | 0,62 | 0,62 | 0,55 | 0,62 | 0,58 | 0,33 | 0,46 | 0,35 | 0,4 | 0,38 |
| -9 | 0,14 | 0,12 | 0,12 | 0,21 | 0,12 | 0,14 | 0,18 | 0,17 | 0,14 | 0,13 | 0,11 | 0,11 |
| -9,5 | 0,08 | 0,08 | 0,06 | 0,06 | 0,07 | 0,09 | 0,01 | 0,01 | 0,02 | 0,02 | 0,04 | 0,02 |

## Supporting Fig 3a and Fig 5a ,5b,5c and 5d

### Mass spectra

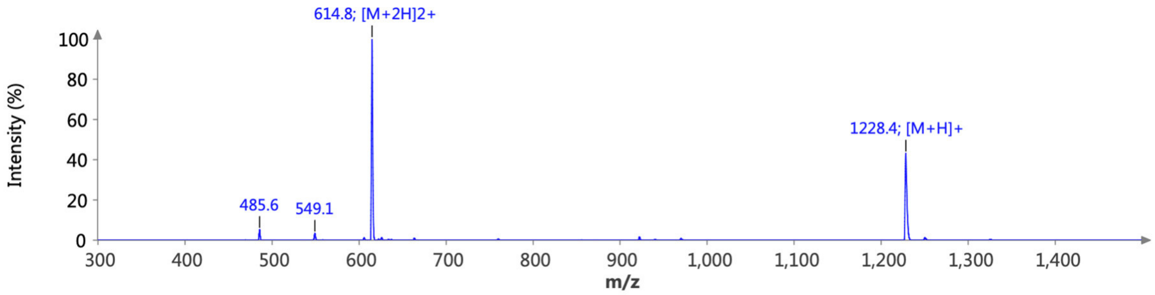


Low resolution ESI-MS(+) of f-MVPIK(Ahx-DOTA)I.

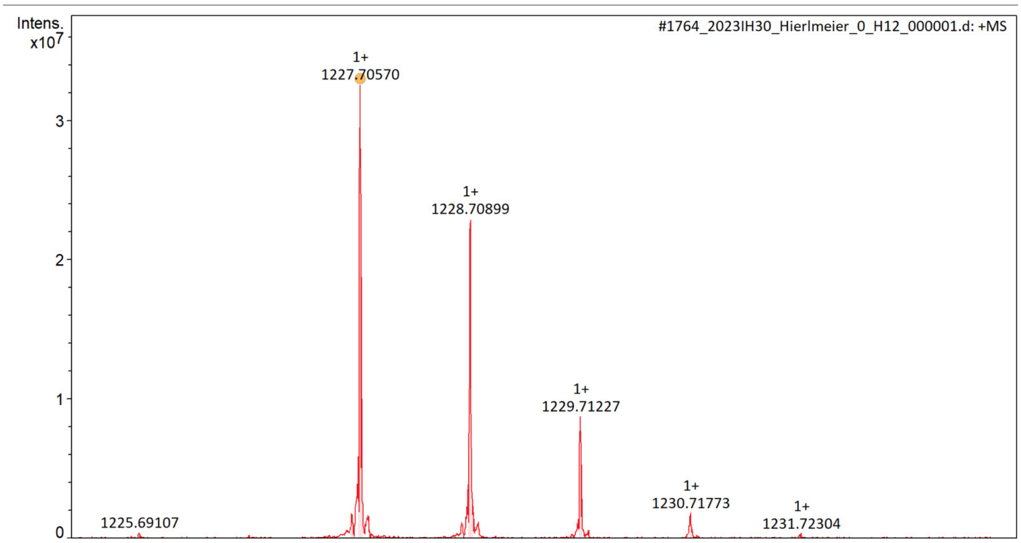


MALDI-TOF of f-MVPIK(Ahx-DOTA)I isotope pattern.

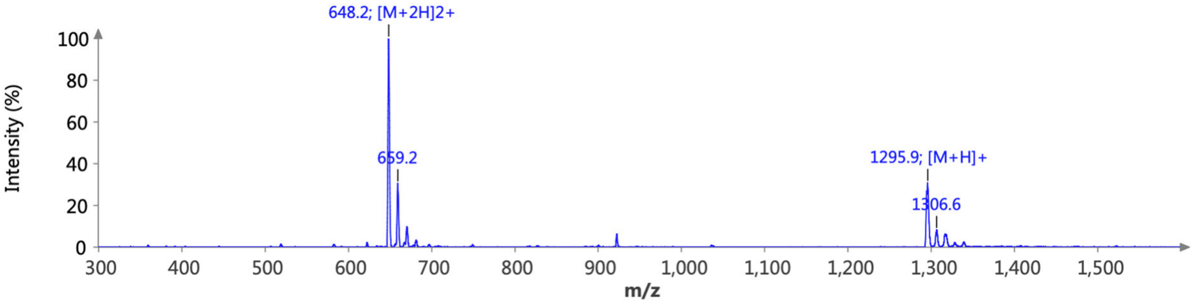


Low resolution ESI-MS(+) of Ga(f-MVPIK(Ahx-DOTA)I).

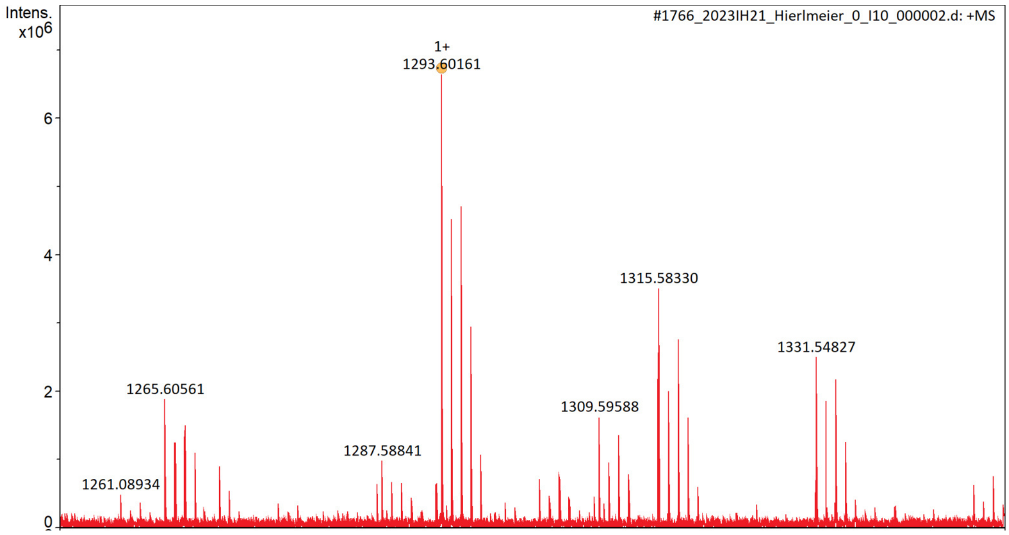


MALDI-TOF of Ga(f-MVPIK(Ahx-DOTA)I) isotope pattern.

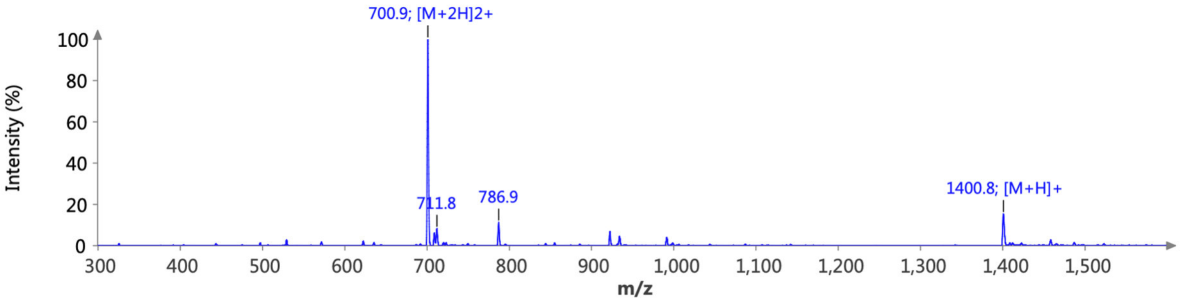


Low resolution ESI-MS(+) of Lu(f-MVPIK(Ahx-DOTA)I)

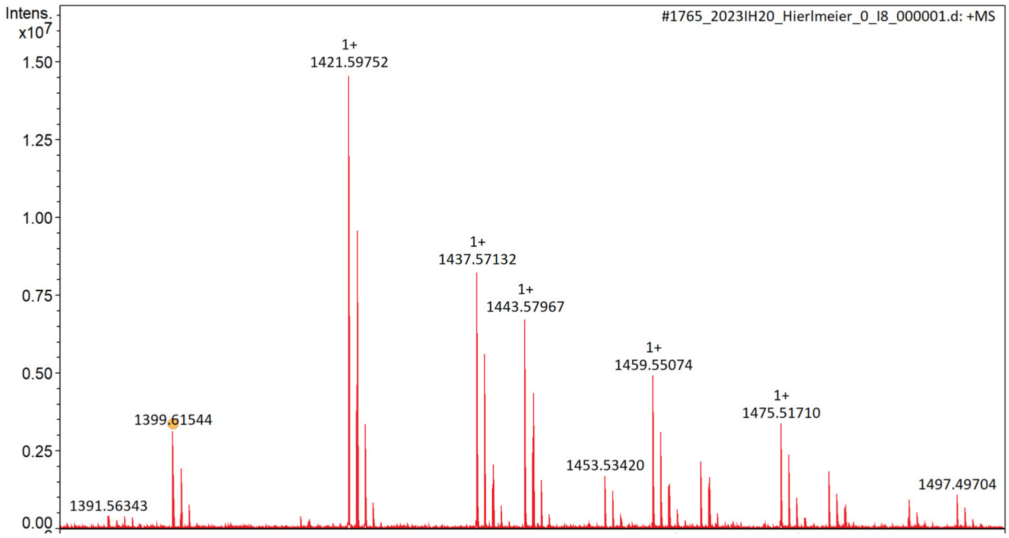


MALDI-TOF of Lu[f-MVPIK(Ahx-DOTA)I].

**Analytical HPLC**

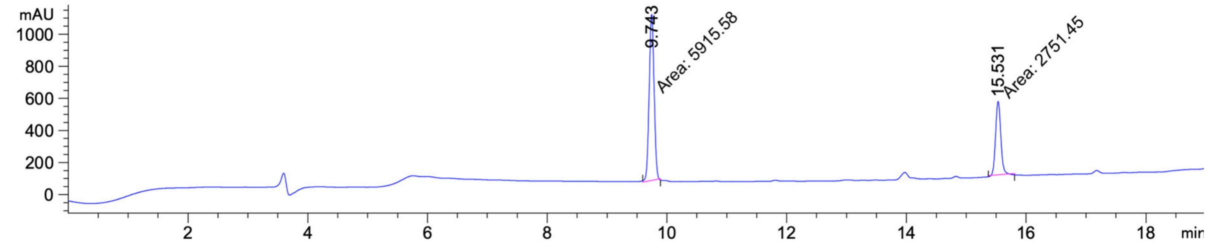


UV/vis RP-PHPLC chromatogram of f-MVPIK(Ahx-DOTA)I (220 nm).

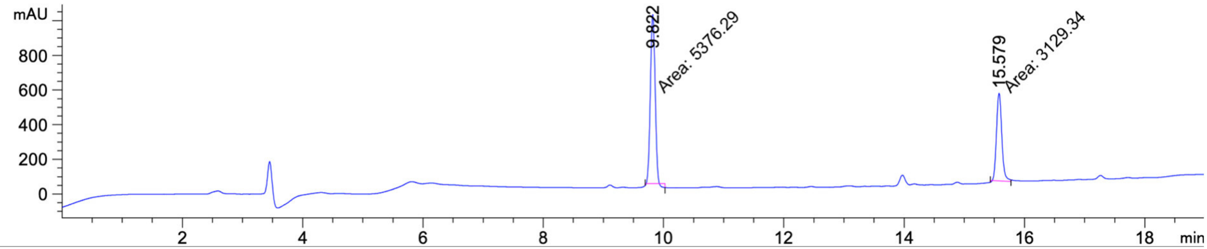


UV/vis RP-PHPLC chromatogram of Ga(f-MVPIK(Ahx-DOTA)I) (220 nm).

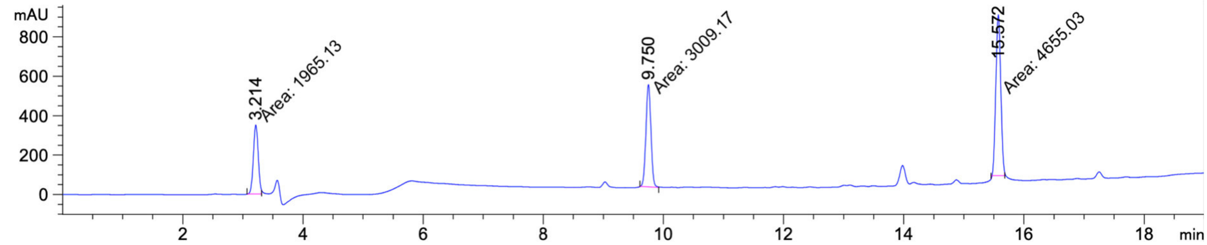


UV/vis RP-PHPLC chromatogram of Lu(f-MVPIK(Ahx-DOTA)I) (220 nm). Peak at 3.214 min = ascorbic acid.

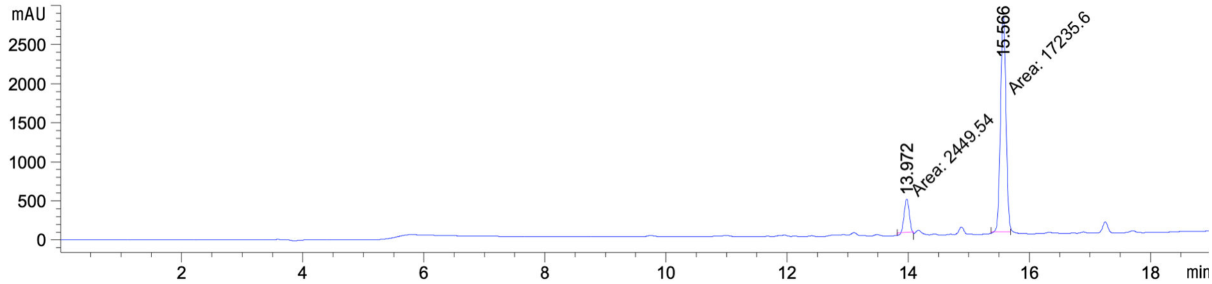


Blank UV/vis RP-PHPLC chromatogram (to show that peaks at 13.9 and 15.5 min are background)

**Radio-HPLC**

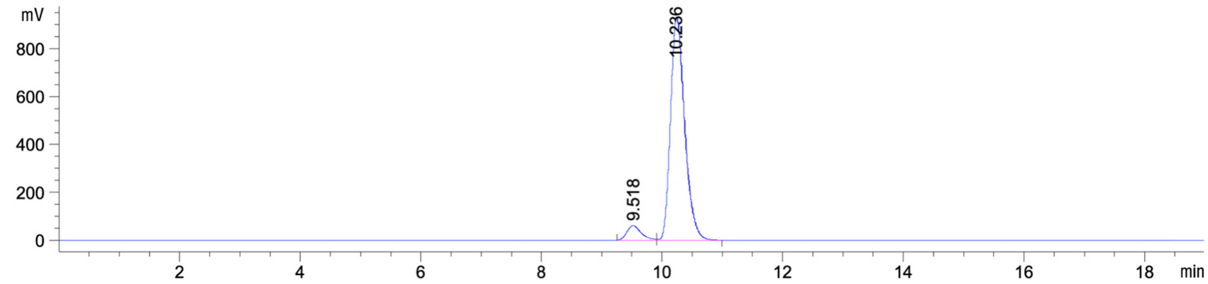


Radio-RP-HPLC chromatogram of [$^{68}$Ga]Ga(f-MVPIK(Ahx-DOTA)I).

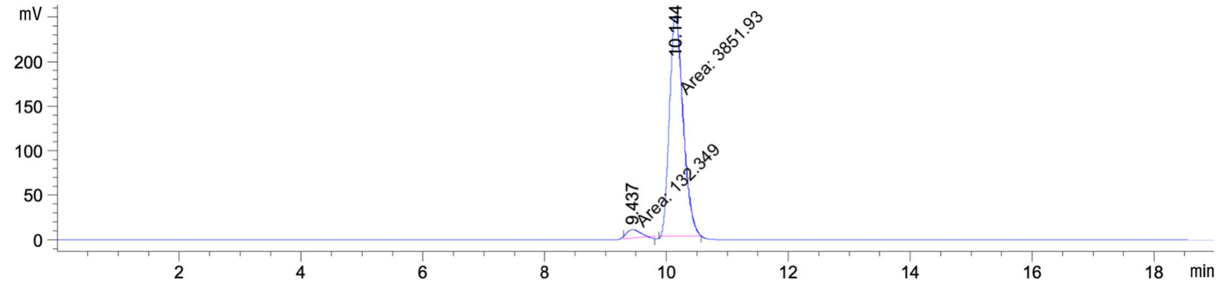


Radio-RP-HPLC chromatogram of [ [$^{177}$Lu]Lu(f-MVPIK(Ahx-DOTA)I).

Supporting Table 1: **Details of all primers used for PCR experiments and Reverse Transcriptase.** Accession numbers and primer sequences which were used for RT-PCR, RT-qPCR and during Reverse Transcriptase.

| Accession No. | Gene | Official Full Name | Oligonucleotide sequence |
|---|---|---|---|
| **Reverse Transcription primers** | | | |
| n.n. | n.n. | CDS (Smart) | 5-AGCAGTGGTAACAACGCAGAGTA CTTTTTTTTTTTTTTTTTTTTTTTTTTTTTTVN -3 |
| n.n. | n.n. | SMART II | 5- AAGCAGTGGTAACAACGCAGAGTA CGCGGG -3 |
| **RT-qPCR primers** | | | |
| **NM_002029.4** | hFPR1 | Homo sapiens formyl peptide receptor 1 (FPR1), transcript variant 2, mRNA | 5-GGGTCCTCTCCTTTGTCGCAGCA-3<br>5-GGCGGGAAGGGCGTGGATCA-3 |
| **NM_001005738** | hFPR2 | Homo sapiens formyl peptide receptor 2 (FPR2), transcript variant 2, mRNA | 5-GCTTGCCGATGTCCATTGTTGCCA-3<br>5-GGCCAGGGAGCTCGTTGGGT-3 |
| **NM_002030.5** | hFPR3 | Homo sapiens formyl peptide receptor 3 | 5-TCAGCGTGCCTATGTCCATCA-3<br>5-ACCACAGCAGCGAAGACACG-3 |
| **NM_002046.7** | hGAPDH | Homo sapiens glyceraldehyde-3-phosphate dehydrogenase | 5-GAAGGTGAAGGTCGGAGTC-3<br>5-GAAGATGGTGATGGGATTTC-3 |

## Standard curves:

**Validation of RT-qPCR primers for all human FPR1, FPR2 and FPR3**: Ct values were plotted against the log of template quantity for each dilution. Representative PCR products for all primer sets were controlled by gel electrophoresis and sequencing for their specificity.

Right: A representative data set of an amplification curve from these dilution series for each primer.

Left: mean standard curves, regression line, and amplification efficiency (n =3, N=3) generated by using a 10-fold serial dilution of a target DNA template starting with 0,1 ng of a purified and sequenced PCR product in t-RNA.

hFPR1

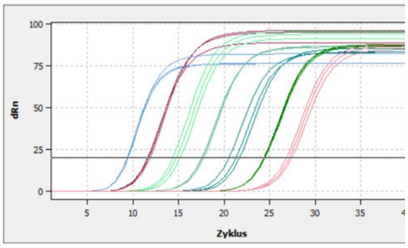


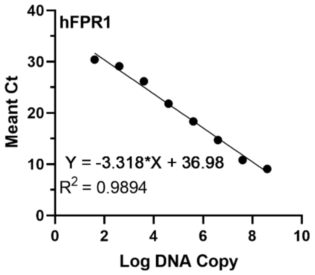

hFPR2

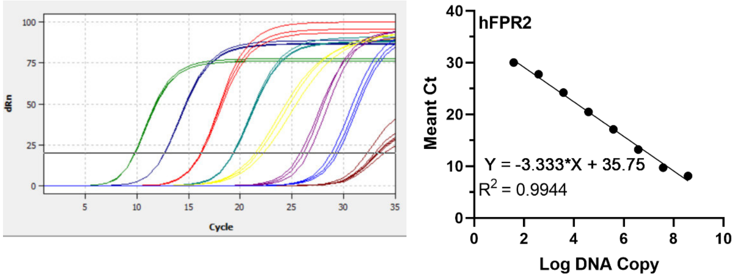


hFPR3

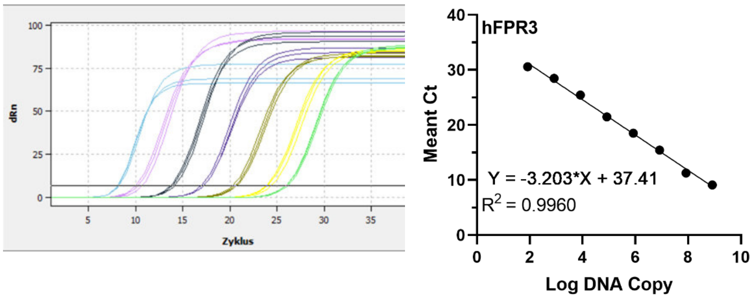


Supplement Fig 4 E: RT-qPCR results of FPR1 expression in U87 and U87 Spheroides (primer hFPR1 , hGAPDH)

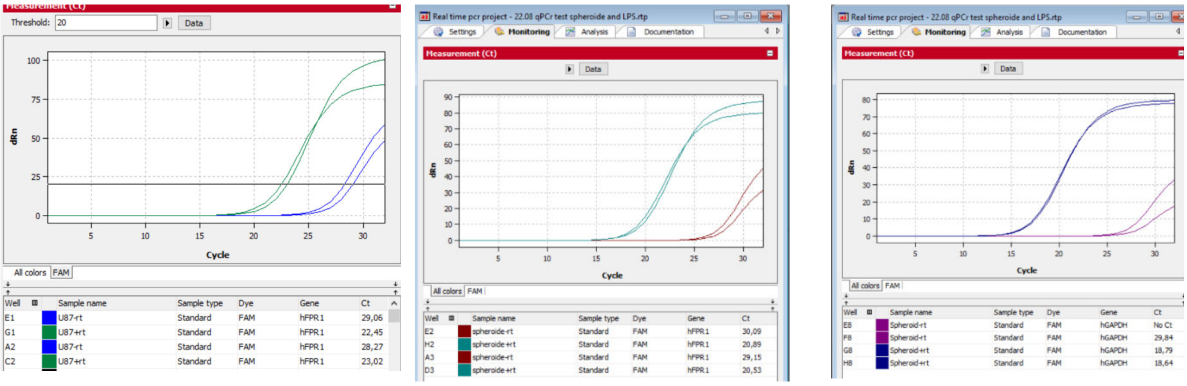

| | | | | | | | |
|---|---|---|---|---|---|---|---|
| **Title:** | **U87 and Spher n=1** | | **Supporting Fig 4e** | | | | |
| **Date/Time:** | **22.08.2023** | **12:15:52** | | | | | |
| **Device:** | **TOptical** | **T-Op2014t 219** | | | | | |
| **Operator:** | **ZA** | | | | | | |
| **Comments:** | | | | | | | |
| **Colors+Dyes:** | **Pos** | **Color Module** | **Dye** | **Gain** | **Meas** | **Refr** | |
| | **1** | **Blue.470.520.1** | **FAM** | **5** | ***** | | |
| **Heated Lid:** | **100°C** | | | | | | |
| **TC Protocol:** | **steps** | **scan** | **°C** | **m:s** | **goto** | **loops** | |
| | **1** | | **95** | **03:00** | | **0** | **0** |
| | **2** | | **95** | **00:05** | | **0** | **0** |
| | **3** | ***** | **65** | **00:10** | | **0** | **0** |
| | **4** | | **66** | **00:10** | | **2** | **31** |
| | **5** | ***** | **60** | **00:15** | | **5** | **35** |
| **Melt active:** | **Yes** | | | | | | |
| **Meas. Repeats** | **3** | | | | | | |
| **Color Comp.:** | | | | | | | |

| **Well** | **Sample name** | **Sample type** | **Dye** | **Gene** | **Ct** | | **Mean Ct** |
|---|---|---|---|---|---|---|---|
| A1 | H2O | NTC | FAM | hFPR1 | | 30,36 | 29,82 |
| B1 | H2O | NTC | FAM | hFPR1 | | 29,27 | 29,82 |
| C1 | gDNA | Positive control | FAM | hFPR1 | | 22,26 | 22,4 |
| D1 | gDNA | Positive control | FAM | hFPR1 | | 22,53 | 22,4 |
| E1 | spheroid-rt | Standard | FAM | hFPR1 | | 29,06 | 28,3 |
| F1 | spheroid-rt | Standard | FAM | hFPR1 | | 27,53 | 28,3 |
| G1 | spheroid+rt | Standard | FAM | hFPR1 | | 22,45 | 22,09 |
| H1 | spheroid+rt | Standard | FAM | hFPR1 | | 21,73 | 22,09 |
| A2 | U87-rt | Standard | FAM | hFPR1 | | 28,27 | 27,59 |
| B2 | U87-rt | Standard | FAM | hFPR1 | | 26,9 | 27,59 |
| C2 | U87+rt | Standard | FAM | hFPR1 | | 23,02 | 21,92 |
| D2 | U87+rt | Standard | FAM | hFPR1 | | 20,81 | 21,92 |
| E2 | spheroide-rt | Standard | FAM | hFPR1 | | 30,09 | 28,25 |
| F2 | spheroide-rt | Standard | FAM | hFPR1 | | 27,09 | 28,25 |
| G2 | spheroide+rt | Standard | FAM | hFPR1 | | 23,56 | 21,83 |
| H2 | spheroide+rt | Standard | FAM | hFPR1 | | 20,89 | 21,83 |
| A3 | spheroide-rt | Standard | FAM | hFPR1 | | 29,15 | 28,25 |
| B3 | spheroide-rt | Standard | FAM | hFPR1 | | 26,69 | 28,25 |
| C3 | spheroide+rt | Standard | FAM | hFPR1 | | 22,35 | 21,83 |
| D3 | spheroide+rt | Standard | FAM | hFPR1 | | 20,53 | 21,83 |
| A8 | H2O | NTC | FAM | hGAPDH | No Ct | | |
| B8 | H2O | NTC | FAM | hGAPDH | No Ct | | |
| C8 | gDNA | Positive control | FAM | hGAPDH | | 20,46 | 20,69 |
| D8 | gDNA | Positive control | FAM | hGAPDH | | 20,93 | 20,69 |
| E8 | Spheroid-rt | Standard | FAM | hGAPDH | No Ct | | |
| F8 | Spheroid-rt | Standard | FAM | hGAPDH | No Ct | | |
| G8 | Spheroid+rt | Standard | FAM | hGAPDH | | 18,79 | 17,52 |
| H8 | Spheroid+rt | Standard | FAM | hGAPDH | | 18,64 | 17,52 |
| A9 | U87-rt | Standard | FAM | hGAPDH | No Ct | | |
| B9 | U87-rt | Standard | FAM | hGAPDH | No Ct | | |
| C9 | U87+rt | Standard | FAM | hGAPDH | | 15,68 | 15 |

| | | | | | | | |
|---|---|---|---|---|---|---|---|
| D9 | U87+rt | Standard | FAM | hGAPDH | | 14,31 | 15 |
| E9 | Spheroid-rt | Standard | FAM | hGAPDH | No Ct | | |
| F9 | Spheroid-rt | Standard | FAM | hGAPDH | No Ct | | |
| H9 | Spheroid+rt | Standard | FAM | hGAPDH | | 16,49 | 17,52 |
| D10 | Spheroid+rt | Standard | FAM | hGAPDH | | 16,19 | 17,52 |

| delta Temp(°C) | delta Time (s) | ramp (°C/s |
|---|---|---|
| 0 | 0 | 5 |
| 0 | 0 | 5 |
| 0 | 0 | 5 |
| 0 | 0 | 5 |
| 1 | 0 | 5 |

| | | | | | | | |
|---|---|---|---|---|---|---|---|
| **Title:** | **U87 and Spher n=2** | | **Supporting Fig 4e** | | | | |
| **Date/Time:** | **01.09.2023** | **11:36:26** | | | | | |
| **Device:** | **TOptical** | **T-Op2014t 219** | | | | | |
| **Operator:** | **ZA** | | | | | | |
| **Comments:** | | | | | | | |
| **Colors+Dyes:** | **Pos** | **Color Module** | **Dye** | **Gain** | **Meas** | **Refr** | |
| | **1** | **Blue.470.520.1** | **FAM** | **5** | ***** | | |
| **Heated Lid:** | **100°C** | | | | | | |
| **TC Protocol:** | **steps** | **scan** | **°C** | **m:s** | **goto** | **loops** | |
| | **1** | | **95** | **03:00** | **0** | **0** | |
| | **2** | | **95** | **00:05** | **0** | **0** | |
| | **3** | ***** | **65** | **00:10** | **0** | **0** | |
| | **4** | | **66** | **00:10** | **2** | **31** | |
| | **5** | ***** | **60** | **00:15** | **5** | **35** | |
| **Melt active:** | **Yes** | | | | | | |
| **Meas. Repeats** | **3** | | | | | | |
| **Color Comp.:** | | | | | | | |

| **Well** | **Sample name** | **Sample type** | **Dye** | **Gene** | **Ct** | | **Mean Ct** |
|---|---|---|---|---|---|---|---|
| A1 | H2O | NTC | FAM | hFPR1 | No Ct | | |
| B1 | H2O | NTC | FAM | hFPR1 | No Ct | | |
| C1 | gDNA | Positive control | FAM | FPR1 | | 22,52 | 22,67 |
| D1 | gDNA | Positive control | FAM | FPR1 | | 22,81 | 22,67 |
| E1 | -rtU87 | Standard | FAM | FPR1 | No Ct | | |
| F1 | -rtU87 | Standard | FAM | FPR1 | No Ct | | |
| G1 | +rtU87 | Standard | FAM | FPR1 | | 25,05 | 25,1 |
| H1 | +rtU87 | Standard | FAM | FPR1 | | 25,15 | 25,1 |
| A2 | -rtU87 sph | Standard | FAM | FPR1 | No Ct | | |
| B2 | -rtU87 sph | Standard | FAM | FPR1 | No Ct | | |
| C2 | +rtU87 sph | Standard | FAM | FPR1 | | 22,28 | 22,43 |
| D2 | +rtU87 sph | Standard | FAM | FPR1 | | 22,59 | 22,43 |
| A10 | H2O | NTC | FAM | hGAPDH | No Ct | | |
| B10 | H2O | NTC | FAM | hGAPDH | No Ct | | |
| C10 | gDNA | Positive control | FAM | hGAPDH | | 21,77 | 21,94 |
| D10 | gDNA | Positive control | FAM | hGAPDH | | 22,12 | 21,94 |
| E10 | -rtU87 | Standard | FAM | GAPDH | No Ct | | |
| F10 | -rtU87 | Standard | FAM | GAPDH | No Ct | | |
| G10 | +rtU87 | Standard | FAM | GAPDH | | 18,79 | 18,76 |
| H10 | +rtU87 | Standard | FAM | GAPDH | | 18,72 | 18,76 |

| delta Temp(°C) | delta Time (s) | ramp (°C/s |
|---|---|---|
| 0 | 0 | 5 |
| 0 | 0 | 5 |
| 0 | 0 | 5 |
| 0 | 0 | 5 |
| 1 | 0 | 5 |

| | | | | | | | |
|---|---|---|---|---|---|---|---|
| **Title:** | **U87 and Sphe** | **n=3** | **Supporting Fig 4e** | | | | |
| **Date/Time:** | **19.09.2023** | **11:56:59** | | | | | |
| **Device:** | **TOptical** | **T-Op2014t 219** | | | | | |
| **Operator:** | **ZA** | | | | | | |
| **Comments:** | | | | | | | |
| **Colors+Dyes:** | **Pos** | **Color Module** | **Dye** | **Gain** | **Meas** | **Refr** | |
| | **1** | **Blue.470.520.1** | **FAM** | | **5** | ***** | |
| **Heated Lid:** | **100°C** | | | | | | |
| **TC Protocol:** | **steps** | **scan** | **°C** | **m:s** | **goto** | **loops** | |
| | **1** | | **95** | **03:00** | | **0** | **0** |
| | **2** | | **95** | **00:05** | | **0** | **0** |
| | **3** | ***** | **65** | **00:10** | | **0** | **0** |
| | **4** | | **66** | **00:10** | | **2** | **31** |
| | **5** | ***** | **60** | **00:15** | | **5** | **35** |
| **Melt active:** | **Yes** | | | | | | |
| **Meas. Repeats** | **3** | | | | | | |
| **Color Comp.:** | | | | | | | |

| **Well** | **Sample name** | **Sample type** | **Dye** | **Gene** | **Ct** | | **Mean Ct** |
|---|---|---|---|---|---|---|---|
| A1 | H2O | NTC | FAM | hFPR1 | No Ct | | |
| B1 | H2O | NTC | FAM | hFPR1 | No Ct | | |
| C1 | gDNA | Positive control | FAM | hFPR1 | | 30,4 | 30,57 |
| D1 | gDNA | Positive control | FAM | hFPR1 | | 30,75 | 30,57 |
| E1 | U87-rt | Standard | FAM | hFPR1 | No Ct | | |
| F1 | U87-rt | Standard | FAM | hFPR1 | No Ct | | |
| G1 | U87+rt | Standard | FAM | hFPR1 | | 25,58 | 25,63 |
| H1 | U87+rt | Standard | FAM | hFPR1 | | 25,7 | 25,63 |
| A2 | U87spher-rt | Standard | FAM | hFPR1 | No Ct | | |
| B2 | U87spher-rt | Standard | FAM | hFPR1 | No Ct | | |
| C2 | U87spher+rt | Standard | FAM | hFPR1 | | 23,69 | 23,85 |
| D2 | U87spher+rt | Standard | FAM | hFPR1 | | 24,01 | 23,85 |
| A5 | H2O | Negative control | FAM | hGAPDH | No Ct | | |
| B5 | H2O | Negative control | FAM | hGAPDH | No Ct | | |
| C5 | gDNA | Positive control | FAM | hGAPDH | | 28,16 | 28,52 |
| D5 | gDNA | Positive control | FAM | hGAPDH | | 28,88 | 28,52 |
| E5 | U87-rt | Standard | FAM | hGAPDH | No Ct | | |
| F5 | U87-rt | Standard | FAM | hGAPDH | No Ct | | |
| G5 | U87+rt | Standard | FAM | hGAPDH | | 19,21 | 18,92 |
| H5 | U87+rt | Standard | FAM | hGAPDH | | 19,29 | 18,92 |
| A6 | U87spheroid1-rt | Standard | FAM | hGAPDH | No Ct | | |
| B6 | U87spheroid1-rt | Standard | FAM | hGAPDH | No Ct | | |
| C6 | U87spheroid1+rt | Standard | FAM | hGAPDH | | 18,38 | 18,57 |
| D6 | U87spheroid1+rt | Standard | FAM | hGAPDH | | 18,77 | 18,57 |

| delta Temp(°C | delta Time (s) | ramp (°C/s |
|---|---|---|
| 0 | 0 | 5 |
| 0 | 0 | 5 |
| 0 | 0 | 5 |
| 0 | 0 | 5 |
| 1 | 0 | 5 |
