# Supplementary material for: Assessing the potential of bacterial signal peptides for radiopharmaceutical applications
Source: Sci Rep. 2025 Sep 12;15:32479. doi: 10.1038/s41598-025-18831-z (PMC12432127; doi:10.1038/s41598-025-18831-z)

Assessing the potential of bacterial signal peptides for radiopharmaceutical applications

Zukaa Al Taleb[1], Ina Hierlmeier[2], Heiko Heilmann[1], Martin Jung[3], Mark Bartholomä[2] and Bernd Bufe[1]

Supplement 1: Characterization of binding affinity and specificity of f-MVPIK(TAMRA)I to FPR receptors.

Representative microscopic images displaying the concentration dependent receptor affinity of the fluorescent labelled f-MVPIK(TAMRA)I probe bound to FPR1, FPR2, FPR3 or mock transfected HEK293T cells.

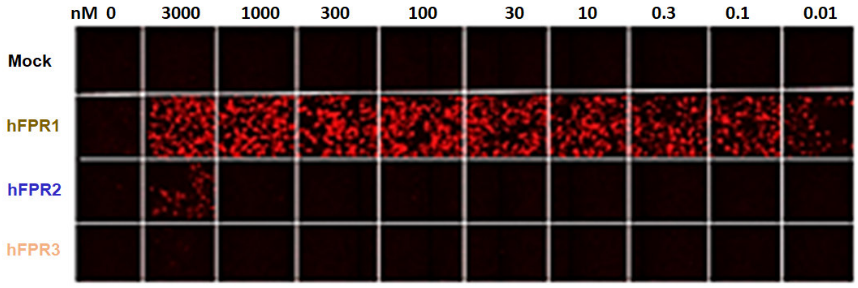

Supplement 2: Expression of FPRs mRNA in U87-MG cancer cell line

The measurement of mRNA expression levels of FPR was performed using RT-qPCR. Absolute quantification of the signals was carried out by normalizing the target genes to the housekeeping reference gene glyceraldehyde-3-phosphate dehydrogenase (GAPDH) and calculated according to specific standard curves (Table 2 in Supplementary original file). The qPCR products were electrophoresed on a 1.5% agarose gel and visualized with Roti-Gelstain (Carl Roth) incorporation under UV light.

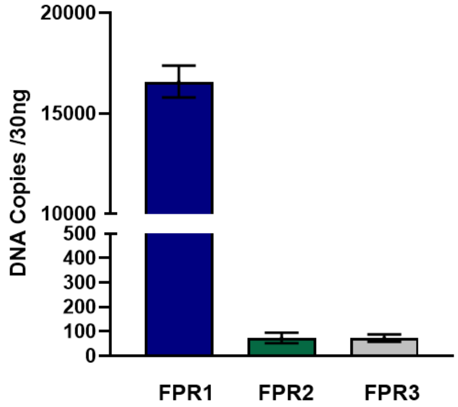

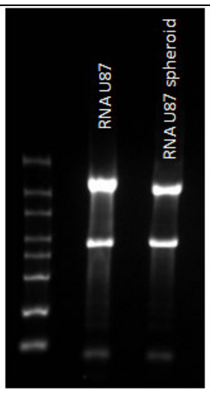

RNA gel image

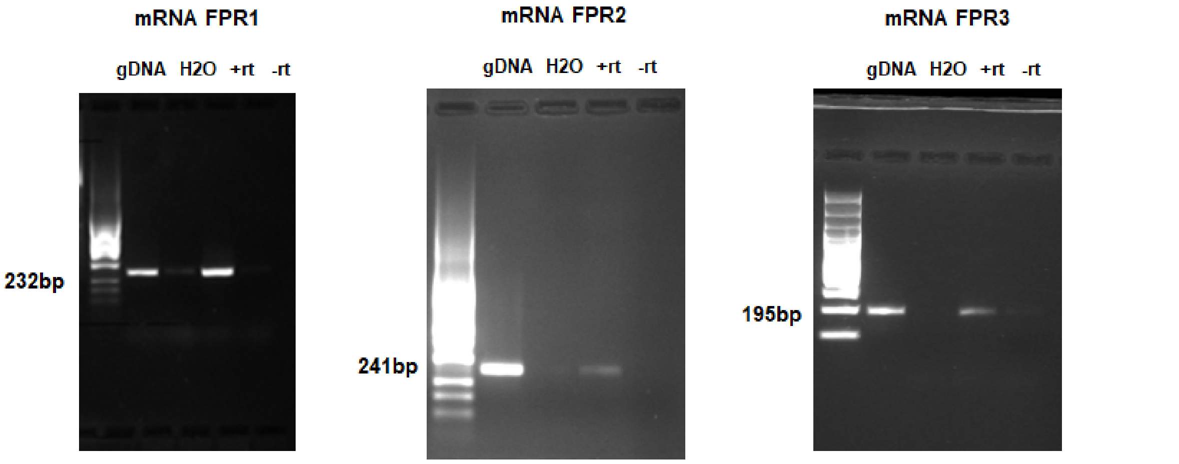

Positive control: genomic DNA (gDNA). Negative control: H_2O. -rt: cDNA without reverse transcriptase
+rt: cDNA with reverse transcriptase

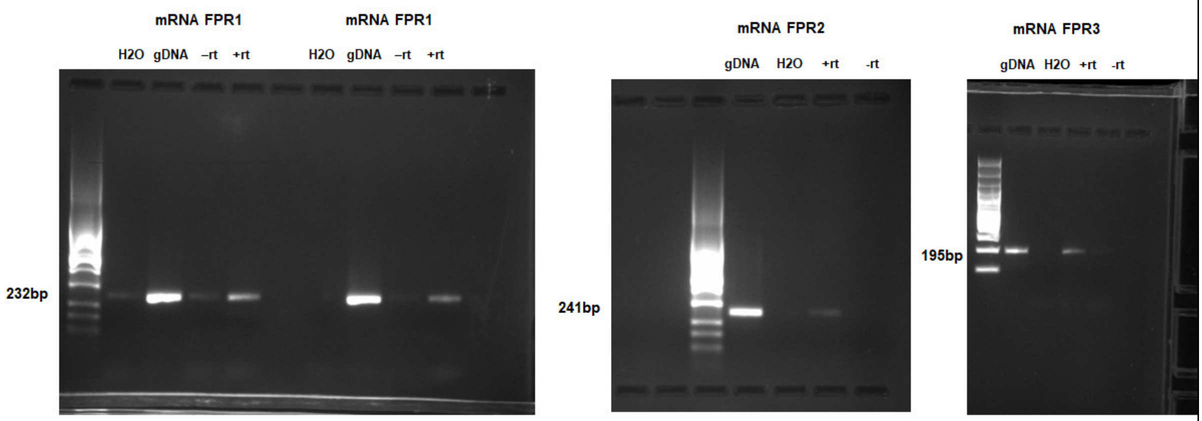

Electrophoresis gel images of some replicates

Supplement 3: U87-MG spheroid formation in 3D cell culture model:

U87-MG glioblastoma cells were suspended at a density optimized for spheroid formation about 2,000 cells/well in Round-bottom 96-well plates (Brand Plates) which used to promote aggregation into a single spheroid per well. To enhance the spheroid formation, wells were coated with 1 mg/ml growth factor collagen Type IV (Thermo Fisher). The Plates were placed in the IncuCyte S3 system to monitor spheroid size and shape at (6 h) intervals time under optimal conditions (37°C, 5% CO_2). Spheroid size ranged between 100–500 µm, depending on initial cell density.

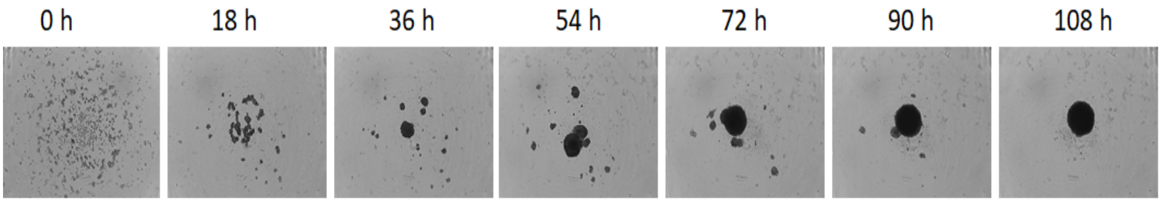

Supplement: Supplementary file 2 — Supplementary Material 2 [file 41598_2025_18831_MOESM2_ESM.pdf]
